# Physiological and Transcriptional Mechanisms Underlying Salt Tolerance in *Quercus acutissima* Regulated by *Funneliformis mosseae*

**DOI:** 10.3390/plants15213450

**Published:** 2026-11-05

**Authors:** Hanlin Liu, Xiaoshuo Jiang, Chang Lu, Yizeng Lu, Dan Liu, Xin Han, Limin Sun

**Affiliations:** 1 Key Laboratory of National Forestry and Grassland Administration for Warm Temperate Forest Ecosystem Conservation and Restoration, Forestry College, Shandong Agricultural University, Tai’an 271018, China; 2 Shandong Provincial Center for Forest and Grassland Germplasm Resources, Jinan 250102, China

**Keywords:** *Quercus acutissima*, salt stress, *Funneliformis mosseae*, mycorrhizal symbiosis

## Abstract

*Quercus acutissima* is an important tree species of coastal shelterbelts in eastern China; however, soil salinization severely constrains its growth and ecological benefits. *Funneliformis mosseae* can enhance plant stress resistance, yet the mechanisms by which it regulates salt tolerance in *Quercus acutissima* remain unclear. In this study, a two-factor pot experiment was conducted on half-sib families of *Quercus acutissima* with four salinity gradients (0‰, 2‰, 4‰, and 6‰, corresponding to 0%, 0.2%, 0.4%, and 0.6% (*w*/*w*) NaCl, respectively) and four inoculant doses (0, 10, 20, and 30 g), with three biological replicates per treatment, and the regulatory pathways were dissected by combining physiological measurements with transcriptome sequencing of functional leaf tissues collected from four selected treatment groups (the uninoculated control, mycorrhizal inoculation alone, salt stress alone, and salt stress combined with mycorrhizal inoculation). The results showed that inoculation with *Funneliformis mosseae* under salt-free conditions induced a slight stress-like response in *Quercus acutissima*; inoculation enhanced salt tolerance only under low-salt conditions (≤2‰ NaCl, mass concentration), with the 30 g high-dose inoculant being the most effective; salt stress promoted excessive root lignification and xylem embolism, whereas symbiosis with *Funneliformis mosseae* alleviated this damage. Successful root colonization by *Funneliformis mosseae* was established in all inoculated treatments, confirming the formation of a functional mycorrhizal symbiosis under both salt-free and salt-stressed conditions. The pentose phosphate pathway, the inositol phosphate–Ca^2+^ signaling pathway, and multiple hormone-related pathways coordinately regulated salt tolerance. This study provides a theoretical basis for cultivating *Quercus acutissima* in salinized soils, offers practical support for afforestation of *Quercus acutissima* on saline lands in the Yellow River Delta, and contributes to improving the quality and efficiency of coastal shelterbelts.

## 1. Introduction

*Quercus acutissima* is a deciduous tree species of the genus *Quercus* [1] and is distributed across China, Japan, Korea, and other countries [2]. This species exhibits strong ecological adaptability and can grow normally in saline–alkali, windblown-sand, and nutrient-poor soils while delivering ecological benefits. As a shelterbelt species along the eastern coast of China, it provides windbreak and sand fixation, protects croplands and improves soil, and offers habitats for a wide range of organisms, thereby maintaining regional biodiversity [3].

Arbuscular mycorrhizal fungi (AMF) are a group of fungi widely distributed in soil that can establish mutualistic symbiosis with the roots of most terrestrial plants, possessing both significant ecological value and agricultural application potential [4]. These fungi can enhance plant water and nutrient uptake efficiency through their extraradical hyphal network, regulate osmotic balance and ion homeostasis, activate antioxidant systems to alleviate oxidative damage, remodel root architecture, and balance endogenous hormone levels, thereby enhancing plant salt tolerance across multiple physiological dimensions [5,6]. Numerous studies abroad have confirmed that AMF can improve ion distribution and antioxidant performance in herbaceous and broad-leaved tree species through their hyphal network, effectively alleviating salt-stress injury [7]. However, existing studies have largely focused on crops and a few fast-growing pioneer woody species, whereas systematic investigations of root anatomical characteristics and transcriptional regulatory pathways in Quercus species remain scarce [8]. Domestic scholars have conducted numerous pot experiments on woody species such as poplar (Populus) and black locust (Robinia pseudoacacia), confirming that AMF can promote the accumulation of osmotic-regulating substances and maintain photosynthetic system stability [9]. However, for coastal shelterbelt oak species such as Q. acutissima, the matching patterns between inoculant doses and salinity gradients, as well as the molecular regulatory mechanisms underlying root xylem remodeling under salt stress, have not been fully elucidated [10]. Funneliformis mosseae, a widely applied dominant arbuscular mycorrhizal fungal species, can comprehensively alleviate the toxic effects of salt stress on woody plants through multiple pathways, including optimizing root nutrient uptake, maintaining intracellular ion balance, strengthening photosynthetic and antioxidant functions, and regulating osmotic metabolism and endogenous hormone signaling [11]. At the mechanistic level, the salt tolerance conferred by mycorrhizal symbiosis is increasingly understood to arise from the integration of plasma-membrane receptor-mediated signaling, hormone crosstalk, and primary carbon metabolism. For example, receptor-like kinase (CrRLK1L)-mediated signaling connects extracellular cues to downstream Ca^2+^ and hormone pathways during stress adaptation [12], while the precise engineering of carbon partitioning underpins the maintenance of redox and energy homeostasis under adverse conditions [13]. These advances provide a conceptual framework for interpreting the Ca^2+^-dependent signaling, hormone crosstalk, and carbon-metabolic responses of Q. acutissima to F. mosseae inoculation under salt stress examined in the present study.

In this study, *Q. acutissima* was used as the experimental material; seedlings were artificially inoculated with *F. mosseae* and subjected to gradient salt-stress treatments to systematically investigate the physiological regulatory effects and transcriptional regulatory mechanisms of *F. mosseae* on salt tolerance in *Q. acutissima*, and to clarify the effects of this symbiotic system on the growth and development of *Q. acutissima* under salt stress [14]. The results can not only provide a practical basis for enhancing the adaptability of *Q. acutissima* in salinized soils, but also offer new insights and technical approaches for improving the quality and efficiency of shelterbelts in coastal saline areas such as the Yellow River Delta, thereby contributing to ecological protection in the Yellow River Basin [15].

## 2. Results

### 2.1. Growth Responses of Q. acutissima to Salinity and F. mosseae Inoculation Dose

#### 2.1.1. Plant Height Growth Response

Under 0‰ NaCl stress, the GC group exhibited the best promoting effect (*p* < 0.05); after 30 days of inoculation, the plant height growth rate of *Q. acutissima* reached 12.23%, which was 42.87% higher than that of the CK group (8.56%). The GA group showed a 30-day plant height growth rate of 10.58%, also higher than that of the CK group. In contrast, the GB group showed a 30-day plant height growth rate of only 7.10%, lower than that of the CK group, indicating an inhibitory effect on plant height growth (*p* < 0.05).

**Figure 1:**
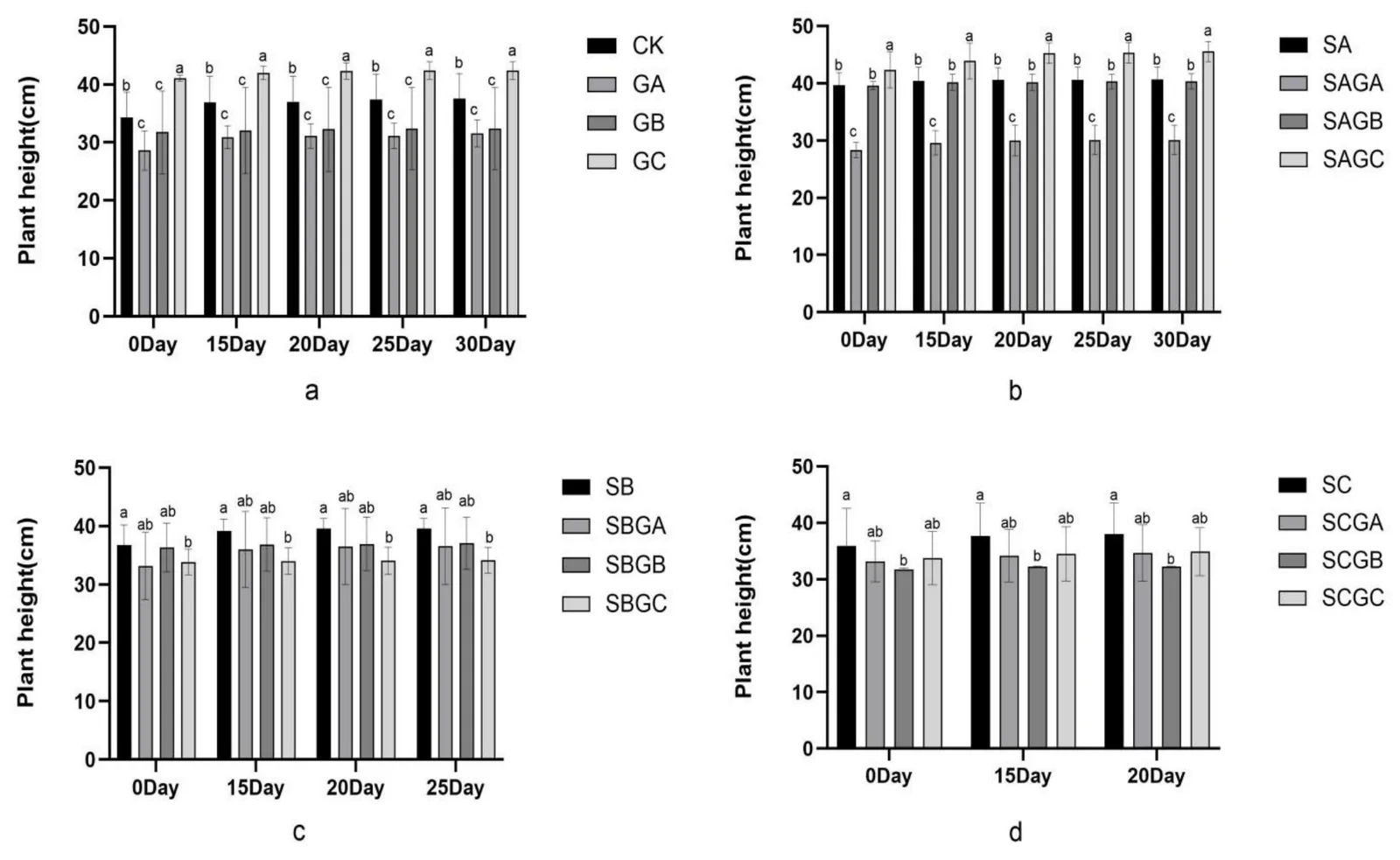
Effects of *Funneliformis mosseae* inoculation on plant height of *Quercus acutissima* under different NaCl concentrations. Note: (**a**) plant height under 0‰ NaCl; (**b**) plant height under 2‰ NaCl; (**c**) plant height under 4‰ NaCl; (**d**) plant height under 6‰ NaCl. Treatment codes (*F. mosseae* inoculum dose, NaCl): CK (0 g, 0‰); GA (10 g, 0‰); GB (20 g, 0‰); GC (30 g, 0‰); SA (0 g, 2‰); SAGA (10 g, 2‰); SAGB (20 g, 2‰); SAGC (30 g, 2‰). Data are shown as mean ± SD (n = 3 biological replicates). Different lowercase letters indicate significant differences among treatments at each sampling time point (one-way or two-way ANOVA followed by Tukey’s multiple-comparison test, *p* < 0.05).

Under 2‰ NaCl conditions, the SA group exhibited a 30-day plant height growth rate of only 2.62%, with growth inhibited by salt stress. The SAGA group showed a 30-day plant height growth rate of 7.00%, which was 167.18% higher than that of the SA group, indicating that mycorrhizal inoculation substantially enhanced the plant growth rate under salt stress (*p* < 0.05). The SAGB group showed a 30-day plant height growth rate of 5.56%, which was 112.21% higher than that of the SA group (*p* < 0.05). However, the SAGC group inoculated with 30 g *F. mosseae* showed a 30-day plant height growth rate of only 1.90%, lower than that of the SA group, with no significant difference between the two groups (*p* > 0.05) (Figure 1).

Under 4‰ and 6‰ NaCl stress, none of the *F. mosseae* doses could alleviate salt injury, and all seedlings died at 7 and 11 days after stress application, respectively; therefore, no valid data on plant height, ground diameter, or leaf number were obtained.

#### 2.1.2. Ground Diameter Growth Response

Under 0‰ NaCl stress, the GC group exhibited a 30-day ground diameter growth rate of 9.83%, which was 15.23% higher than that of the CK group (13.00%), showing the best thickening effect (*p* < 0.05). The GA group showed a 30-day ground diameter growth rate of 13.87%, with no significant difference from the CK group (*p* > 0.05). In contrast, the GB group showed a ground diameter growth rate of only 2.88%, significantly lower than that of the CK group, inhibiting ground diameter thickening (*p* < 0.05).

**Figure 2:**
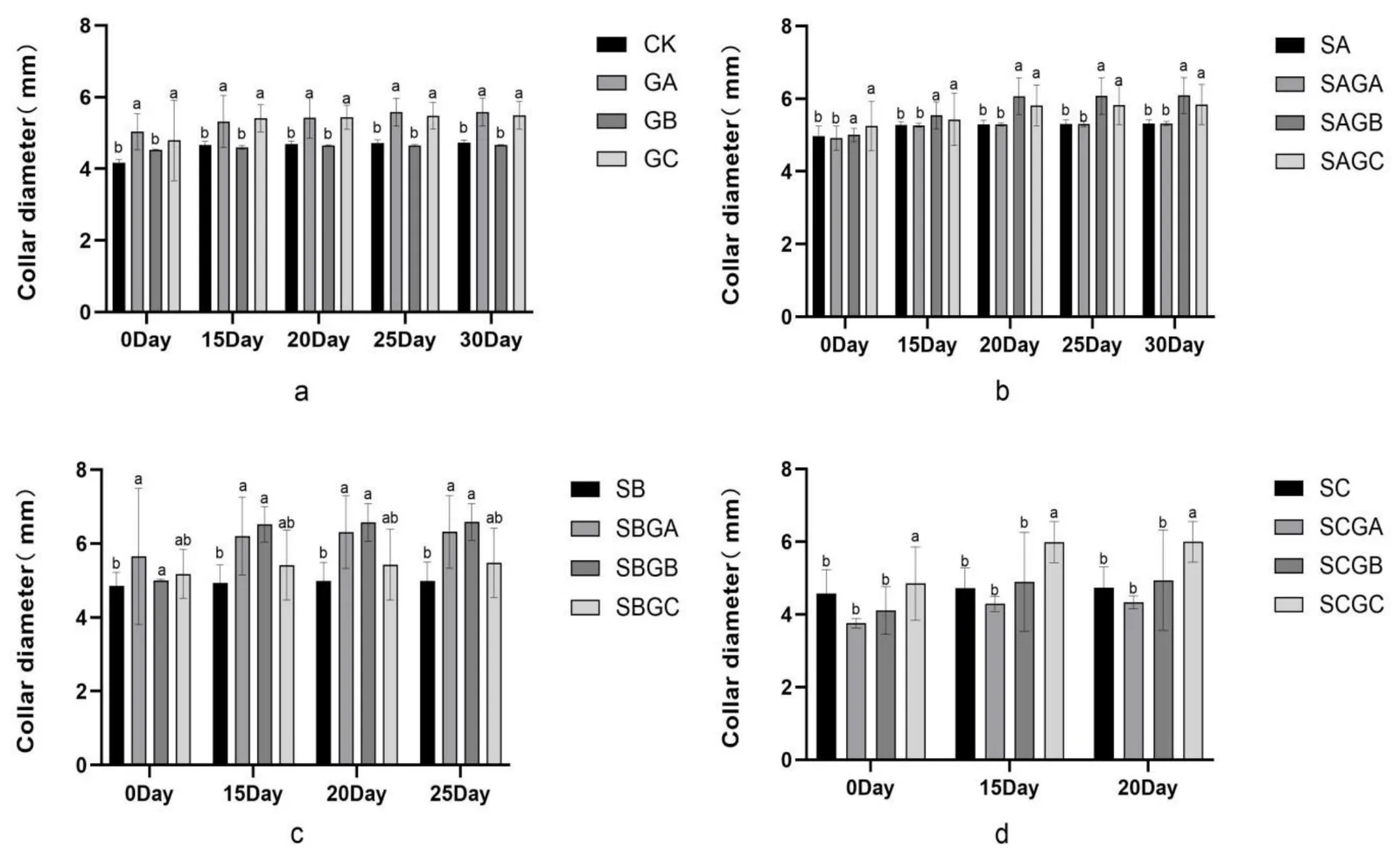
Effects of *Funneliformis mosseae* inoculation on ground diameter of *Quercus acutissima* under different NaCl concentrations. Note: (**a**) ground diameter under 0‰ NaCl; (**b**) ground diameter under 2‰ NaCl; (**c**) ground diameter under 4‰ NaCl; (**d**) ground diameter under 6‰ NaCl. Treatment codes (*F. mosseae* inoculum dose, NaCl): CK (0 g, 0‰); GA (10 g, 0‰); GB (20 g, 0‰); GC (30 g, 0‰); SA (0 g, 2‰); SAGA (10 g, 2‰); SAGB (20 g, 2‰); SAGC (30 g, 2‰). Data are shown as mean ± SD (n = 3 biological replicates). Different lowercase letters indicate significant differences among treatments at each sampling time point (one-way or two-way ANOVA followed by Tukey’s multiple-comparison test, *p* < 0.05).

Under 2‰ NaCl conditions, salt stress significantly inhibited ground diameter growth (*p* < 0.01), with the SA group exhibiting a 30-day ground diameter growth rate of only 9.85%. The SAGC group showed a 30-day ground diameter growth rate of 32.24%, which was 227.31% higher than that of the SA group, indicating an extremely significant thickening effect (*p* < 0.01). The SAGB group showed a growth rate of 14.53%, which was 47.51% higher than that of the SA group, with a significant difference (*p* < 0.05). In contrast, the SAGA group showed a growth rate of only 2.52%, significantly lower than that of the SA group, aggravating the inhibitory effect of salt on ground diameter (*p* < 0.05) (Figure 2).

#### 2.1.3. Leaf Number Growth Response

Under 0‰ NaCl stress, there was no significant difference in leaf number increase between any of the *F. mosseae* -inoculated groups and the CK group (*p* > 0.05). The GA group showed a 30-day leaf growth rate of 15.00%, the GB group showed a rate of 3.23%, and the GC group produced no new leaves; none of the groups exhibited a significant difference from the CK group in leaf number increase.

**Figure 3:**
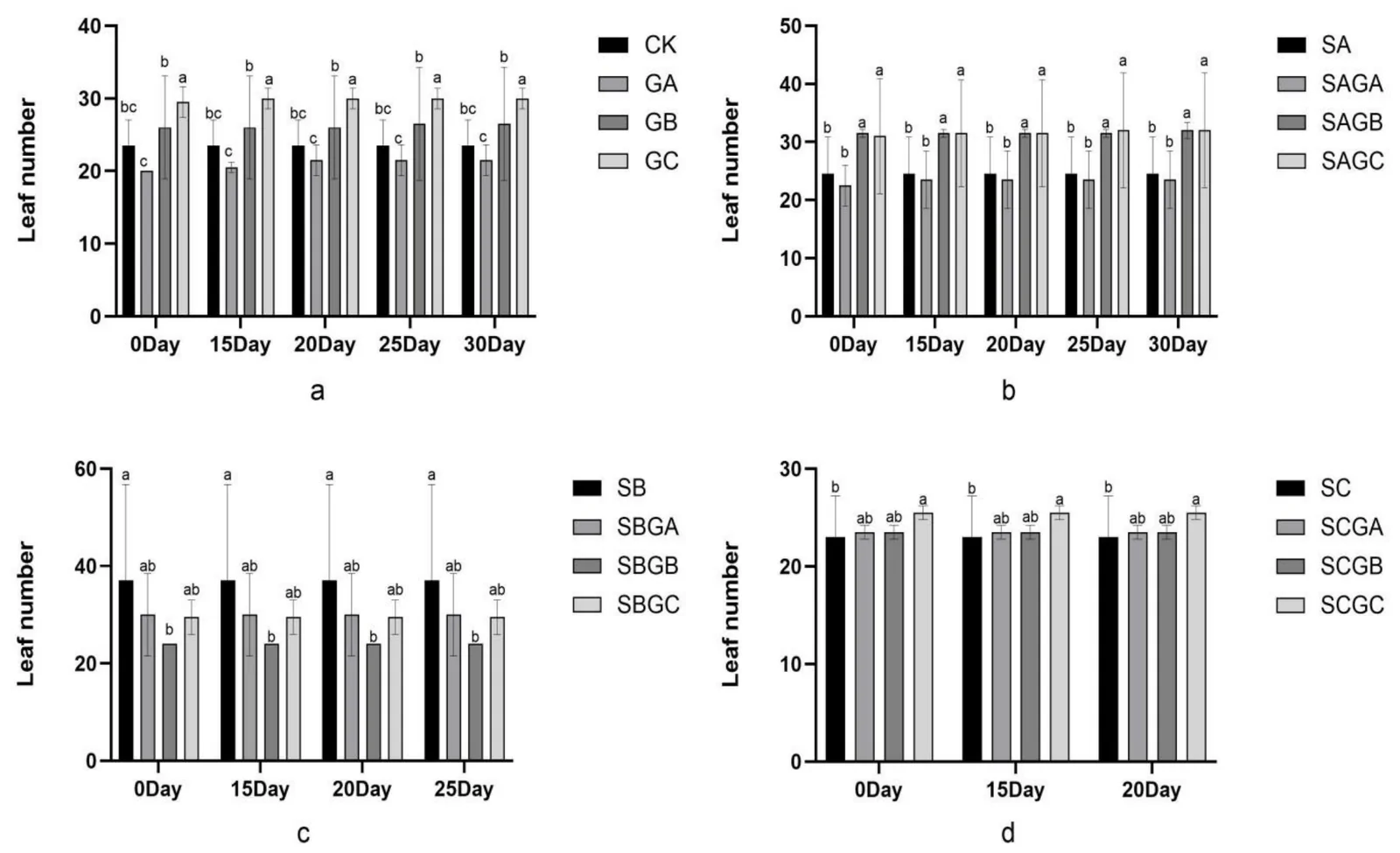
Effects of *Funneliformis mosseae* inoculation on leaf number of *Quercus acutissima* under different NaCl concentrations. Note: (**a**) leaf number under 0‰ NaCl; (**b**) leaf number under 2‰ NaCl; (**c**) leaf number under 4‰ NaCl; (**d**) leaf number under 6‰ NaCl. Treatment codes (*F. mosseae* inoculum dose, NaCl): CK (0 g, 0‰); GA (10 g, 0‰); GB (20 g, 0‰); GC (30 g, 0‰); SA (0 g, 2‰); SAGA (10 g, 2‰); SAGB (20 g, 2‰); SAGC (30 g, 2‰). Data are shown as mean ± SD (n = 3 biological replicates). Different lowercase letters indicate significant differences among treatments at each sampling time point (one-way or two-way ANOVA followed by Tukey’s multiple-comparison test, *p* < 0.05).

Under 2‰ NaCl conditions, the SA group showed a 30-day leaf growth rate of 0%. The SAGB and SAGC groups showed leaf growth rates of 3.13% and 4.17%, respectively, which were slightly higher than those of the SA group, but the differences were not significant (*p* > 0.05). The SAGA group also showed a leaf growth rate of 0%, with no difference from the SA group (Figure 3).

### 2.2. Physiological Responses of Q. acutissima to Salinity and Inoculation Dose

Owing to the lethal effects of high-concentration salt stress, all *Q. acutissima* seedlings in the 4‰ NaCl (SB) treatment group died at 7 days after the application of salt stress, and all seedlings in the 6‰ NaCl (SC) treatment group died at 11 days after the application of salt stress; consequently, the available valid physiological observation data were extremely limited. Therefore, for POD activity and all subsequent physiological indicators, this study focused on and described only the treatment groups under the 0‰ and 2‰ NaCl gradients.

#### 2.2.1. POD Activity

**Figure 4:**
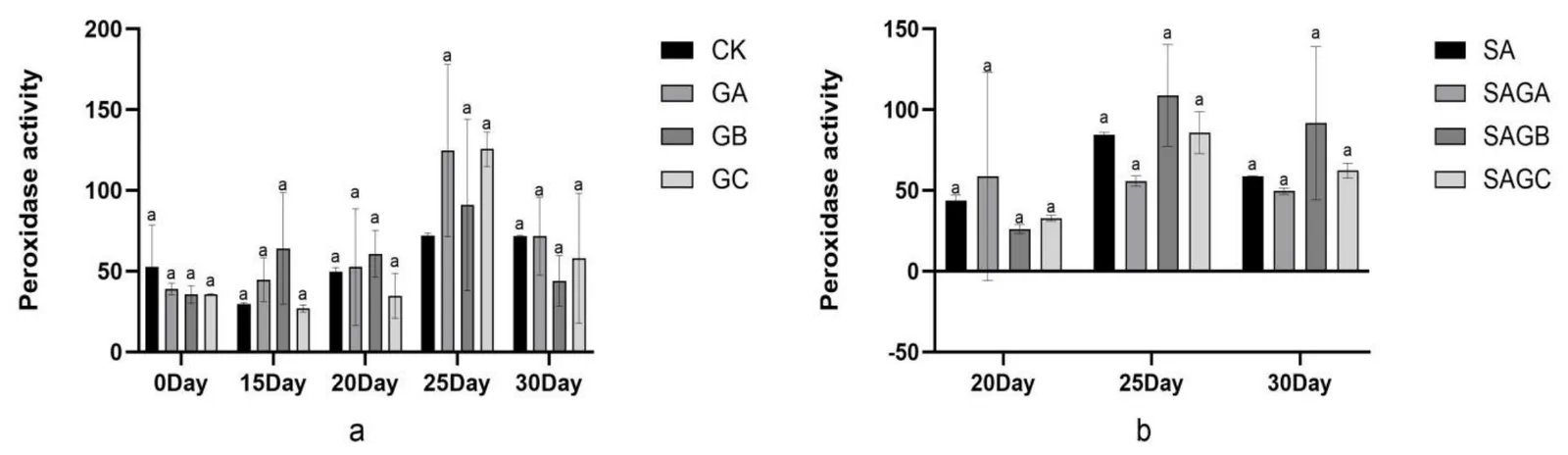
Effects of *Funneliformis mosseae* inoculation on POD activity of *Quercus acutissima* under 0‰ and 2‰ NaCl. Note: (**a**) POD activity under 0‰ NaCl; (**b**) POD activity under 2‰ NaCl. Treatment codes (*F. mosseae* inoculum dose, NaCl): CK (0 g, 0‰); GA (10 g, 0‰); GB (20 g, 0‰); GC (30 g, 0‰); SA (0 g, 2‰); SAGA (10 g, 2‰); SAGB (20 g, 2‰); SAGC (30 g, 2‰). Data are shown as mean ± SD (n = 3 biological replicates). Different lowercase letters indicate significant differences among treatments at each sampling time point (one-way or two-way ANOVA followed by Tukey’s multiple-comparison test, *p* < 0.05).

Under 0‰ NaCl conditions, POD activity did not increase monotonically with the inoculant dose; instead, high and low doses exerted opposite effects: the GA and GC groups increased to 88.5 and 86.5 U/(g·min), respectively, representing increases of 24.65% and 21.83% compared with the CK group (71.0 U/(g·min)) (*p* < 0.05); in contrast, the GB group decreased to 33.0 U/(g·min), representing a decrease of 53.52% compared with the CK group (*p* < 0.05). After transfer to 2‰ NaCl conditions, POD activity in the SA group decreased by 16.90% compared with the CK group; at this point, the SAGC group inoculated with 30 g recovered to 65.5 U/(g·min), representing an increase of 11.02% compared with the SA group (*p* < 0.05), whereas the SAGB group inoculated with 20 g showed no significant difference from the SA group (*p* > 0.05), and the SAGA group inoculated with 10 g decreased by 13.56% compared with the SA group (*p* < 0.05) (Figure 4). The relatively large standard deviations of the SA and SAGC groups under 2‰ NaCl reflect the high intrinsic biological variability in POD activity among individual seedlings within the same treatment; no outliers were removed from the dataset, and the significance letters were assigned strictly according to Tukey’s test results.

#### 2.2.2. MDA Content

**Figure 5:**
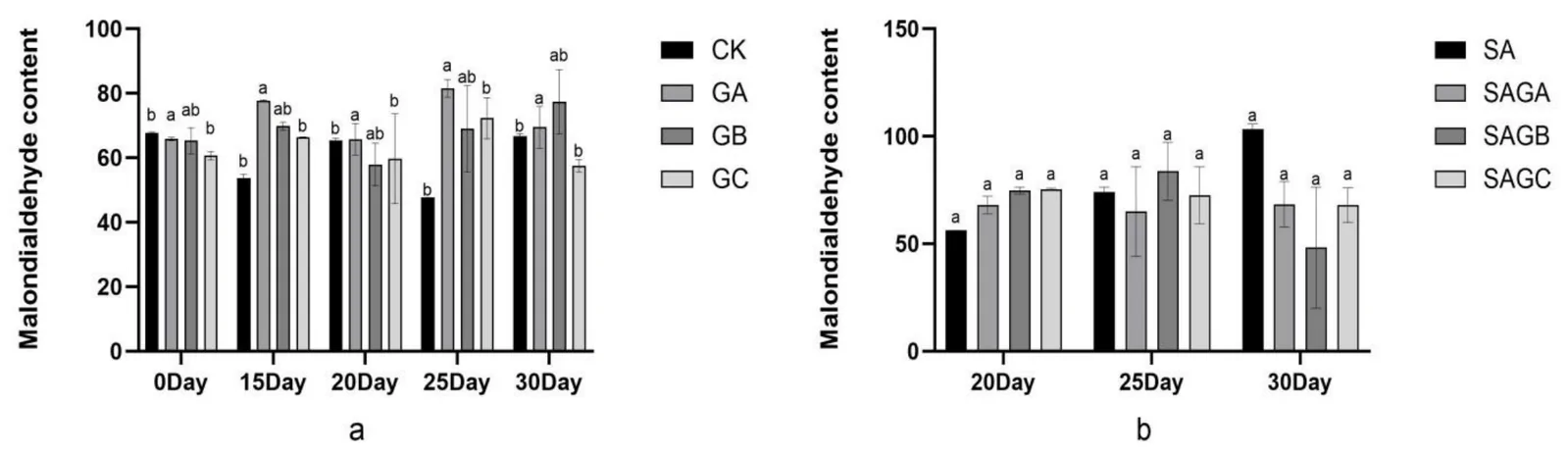
Effects of *Funneliformis mosseae* inoculation on MDA content of *Quercus acutissima* under 0‰ and 2‰ NaCl. Note: (**a**) MDA content under 0‰ NaCl; (**b**) MDA content under 2‰ NaCl. Treatment codes (*F. mosseae* inoculum dose, NaCl): CK (0 g, 0‰); GA (10 g, 0‰); GB (20 g, 0‰); GC (30 g, 0‰); SA (0 g, 2‰); SAGA (10 g, 2‰); SAGB (20 g, 2‰); SAGC (30 g, 2‰). Data are shown as mean ± SD (n = 3 biological replicates). Different lowercase letters indicate significant differences among treatments at each sampling time point (one-way or two-way ANOVA followed by Tukey’s multiple-comparison test, *p* < 0.05).

The changes in MDA content more clearly revealed the correspondence between the inoculant dose and membrane lipid peroxidation damage. Under 0‰ NaCl conditions, the MDA contents in the GC and GA groups decreased to 0.95 and 1.02 nmol/(g·FW), respectively, which were 25.78% and 20.31% lower than those of the CK group (1.28 nmol/(g·FW)), and both were significantly lower than the control (*p* < 0.05). The GB group showed a content of 1.31 nmol/(g·FW), which differed little from the CK group (+2.34%, *p* > 0.05). Under 2‰ NaCl conditions, MDA content in the SA group increased to 1.86 nmol/(g·FW), which was 45.31% higher than that of the CK group, indicating markedly aggravated membrane damage. In the group inoculated with 30 g (SAGC), the MDA content decreased to 1.42 nmol/(g·FW), which was 23.66% lower than that of the SA group (*p* < 0.05); the SAGB group showed a decrease of 9.14% (*p* > 0.05), whereas the SAGA group showed an increase of 8.06% (*p* < 0.05), further amplifying the peroxidation damage (Figure 5).

#### 2.2.3. Proline Content

**Figure 6:**
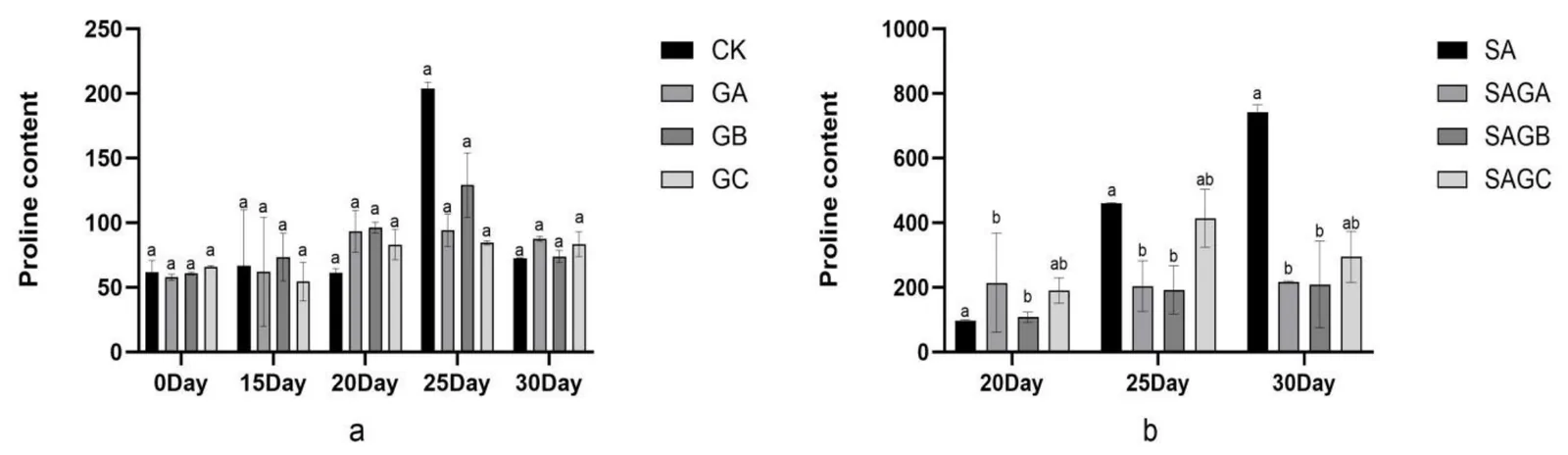
Effects of *Funneliformis mosseae* inoculation on proline (PRO) content of *Quercus acutissima* under 0‰ and 2‰ NaCl. Note: (**a**) PRO content under 0‰ NaCl; (**b**) PRO content under 2‰ NaCl. Treatment codes (*F. mosseae* inoculum dose, NaCl): CK (0 g, 0‰); GA (10 g, 0‰); GB (20 g, 0‰); GC (30 g, 0‰); SA (0 g, 2‰); SAGA (10 g, 2‰); SAGB (20 g, 2‰); SAGC (30 g, 2‰). Data are shown as mean ± SD (n = 3 biological replicates). Different lowercase letters indicate significant differences among treatments at each sampling time point (one-way or two-way ANOVA followed by Tukey’s multiple-comparison test, *p* < 0.05).

The accumulation of proline was generally synchronized with the osmotic adjustment demand. Under salt-free conditions, the proline contents in the GC and GA groups decreased to 22.3 and 24.5 μg/(g·FW), respectively, which were 22.03% and 14.34% lower than that of the CK group (28.6 μg/(g·FW)), and both were significantly lower than the control (*p* < 0.05). The GB group showed a content of 29.1 μg/(g·FW), which was 1.75% higher than that of the CK group, with no significant difference (*p* > 0.05). Under 2‰ NaCl conditions, proline content in the SA group rapidly accumulated to 42.8 μg/(g·FW), which was 49.65% higher than that of the CK group. In the SAGC group, the content decreased to 33.6 μg/(g·FW), which was 21.50% lower than that of the SA group (*p* < 0.05); the SAGB group showed a decrease of 8.64% compared with the SA group (*p* > 0.05), whereas the SAGA group showed an increase of 6.78%, resulting in excessive proline accumulation (*p* < 0.05) (Figure 6).

#### 2.2.4. Leaf Relative Electrical Conductivity (EC)

**Figure 7:**
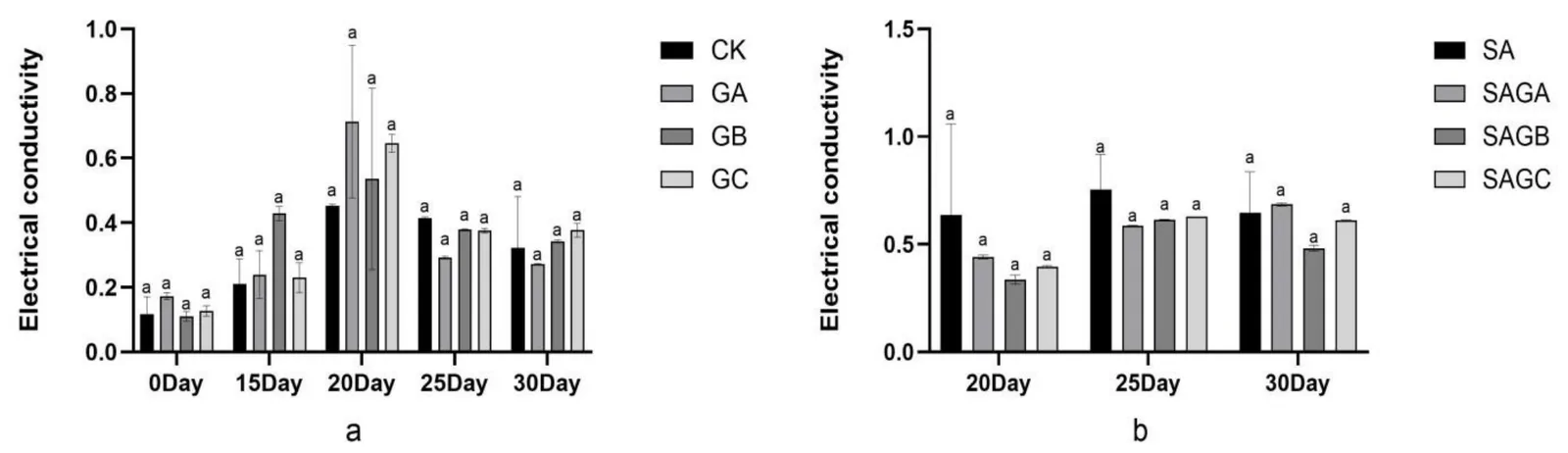
Effects of *Funneliformis mosseae* inoculation on EC value of *Quercus acutissima* under 0‰ and 2‰ NaCl. Note: (**a**) EC value under 0‰ NaCl; (**b**) EC value under 2‰ NaCl. Treatment codes (*F. mosseae* inoculum dose, NaCl): CK (0 g, 0‰); GA (10 g, 0‰); GB (20 g, 0‰); GC (30 g, 0‰); SA (0 g, 2‰); SAGA (10 g, 2‰); SAGB (20 g, 2‰); SAGC (30 g, 2‰). Data are shown as mean ± SD (n = 3 biological replicates). Different lowercase letters indicate significant differences among treatments at each sampling time point (one-way or two-way ANOVA followed by Tukey’s multiple-comparison test, *p* < 0.05).

The response of leaf relative electrical conductivity to the inoculant dose was not monotonic. Under 0‰ NaCl conditions, only the GA group (10 g) reduced EC; at 30 d, the GA group showed a value of 0.272, which was 15.49% lower than that of the CK group (0.322). In contrast, the EC values of the GB and GC groups increased to 0.342 and 0.377, representing increases of 6.31% and 17.02% compared with the CK group, respectively, and showed an upward trend with increasing dose (*p* > 0.05). Under 2‰ NaCl conditions, leaf EC was significantly elevated, with the SA group reaching 0.647 at 30 d, which was 100.83% higher than that of the CK group, indicating aggravated cell membrane damage. At this concentration, the 20 g dose exerted the strongest inhibition on EC: the SAGB group showed an EC of 0.482, which was 25.45% lower than that of the SA group, and the SAGC group showed an EC of 0.611 (5.56% lower than that of the SA group); in contrast, the SAGA group increased to 0.685 (5.91% higher than that of the SA group). However, none of the above differences reached statistical significance (*p* > 0.05) (Figure 7).

#### 2.2.5. Chlorophyll Content

**Figure 8:**
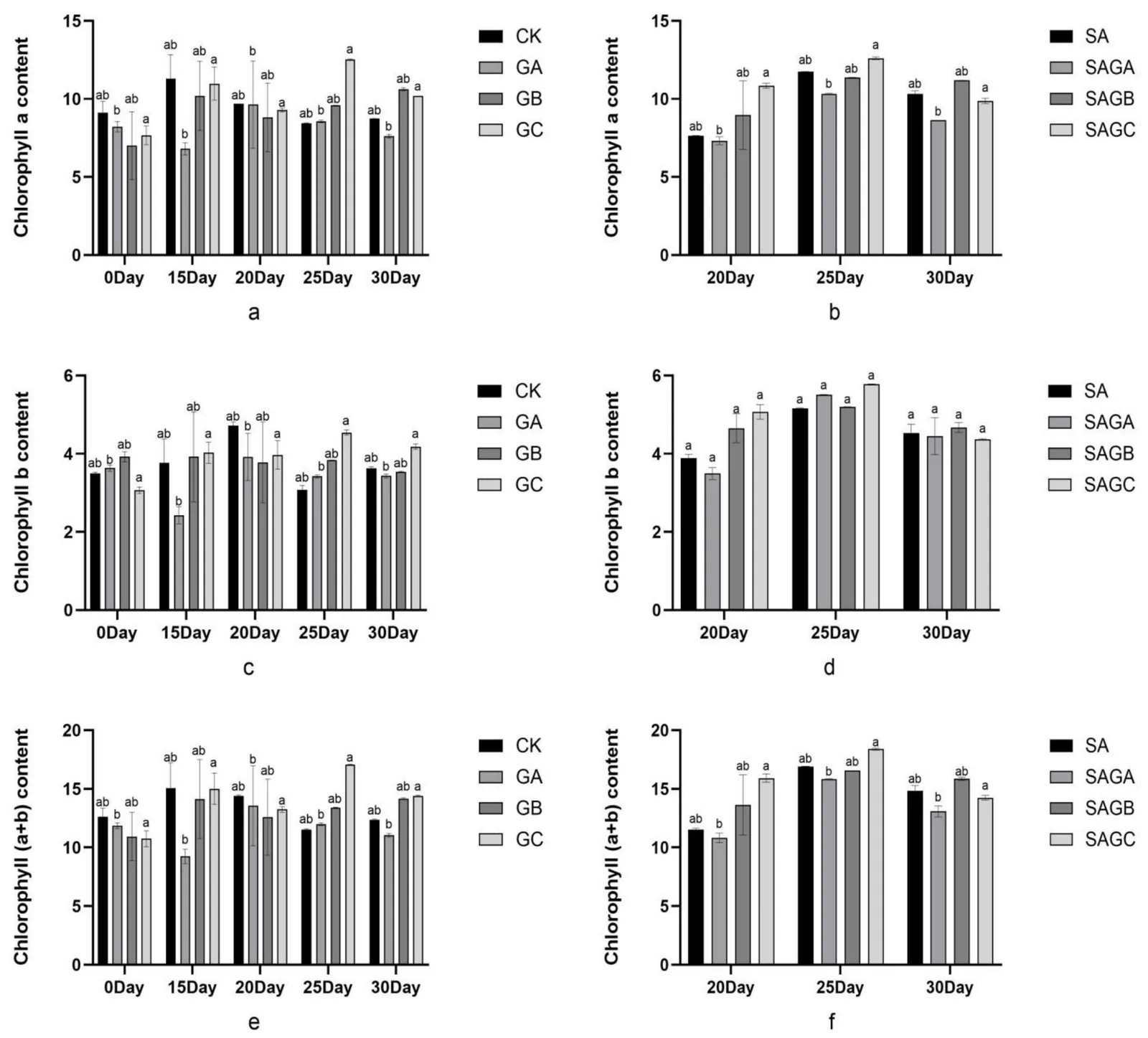
Effects of *Funneliformis mosseae* inoculation on chlorophyll content of *Quercus acutissima* under 0‰ and 2‰ NaCl. Note: (**a**) chlorophyll a content under 0‰ NaCl; (**b**) chlorophyll a content under 2‰ NaCl; (**c**) chlorophyll b content under 0‰ NaCl; (**d**) chlorophyll b content under 2‰ NaCl; (**e**) total chlorophyll content under 0‰ NaCl; (**f**) total chlorophyll content under 2‰ NaCl. Treatment codes (*F. mosseae* inoculum dose, NaCl): CK (0 g, 0‰); GA (10 g, 0‰); GB (20 g, 0‰); GC (30 g, 0‰); SA (0 g, 2‰); SAGA (10 g, 2‰); SAGB (20 g, 2‰); SAGC (30 g, 2‰). Data are shown as mean ± SD (n = 3 biological replicates). Different lowercase letters indicate significant differences among treatments at each sampling time point (one-way or two-way ANOVA followed by Tukey’s multiple-comparison test, *p* < 0.05).

Chlorophyll content generally increased with increasing inoculation dose. Under 0‰ NaCl conditions, the 10, 20, and 30 g doses all significantly increased pigment content (*p* < 0.05). At 30 d, the chlorophyll a, chlorophyll b, and total chlorophyll contents in the GC group were 10.685, 3.531, and 14.216, respectively, with chlorophyll a and total chlorophyll increasing by 22.32% and 14.74% compared with the CK group (8.735, 3.657, and 12.392, respectively). The total chlorophyll contents in the GB and GA groups decreased by 12.04% and 0.57% compared with the CK group, respectively, and the 30 g dose exhibited the best effect. Under 2‰ NaCl conditions, the chlorophyll a, chlorophyll b, and total chlorophyll contents in the SA group were 10.457, 4.688, and 15.145, respectively, with total chlorophyll increasing by only 2.04% compared with the CK group, indicating marked pigment damage. The corresponding values in the SAGC group were 11.193, 4.759, and 15.952, respectively, with chlorophyll a, chlorophyll b, and total chlorophyll increasing by 7.04%, 1.51%, and 5.33% compared with the SA group, respectively; the total chlorophyll contents in the SAGB and SAGA groups decreased by 4.00% and 3.93% compared with the SA group, respectively (Figure 8).

### 2.3. Root Responses of Q. acutissima to Salinity and Inoculation Dose

#### 2.3.1. Root Architectural Indicators

Under 0‰ NaCl conditions, inoculation with *F. mosseae* generally promoted root development; most of the six indicators (total root length, root projected area, root volume, mean root diameter, total root number, and number of root forks) were higher than those of the CK group at 15 d, and the promoting effect increased with increasing dose, being most pronounced in the GC (30 g) group. At 15 d, the GC group showed a total root length of 1477.4 cm, a root projected area of 90.2 cm^2^, a root volume of 4.33 cm^3^, and a number of root forks of 4255, representing increases of 52.2%, 64.7%, 75.4%, and 80.3% compared with the CK group (970.5 cm, 54.8 cm^2^, 2.47 cm^3^, and 2360), respectively. The GA (10 g) group showed a total root length of 1321.3 cm, and the GB (20 g) group 1156.9 cm, both higher than that of the CK group; the differences in mean root diameter and total root number among groups were minor. By 30 d, the root indicators of all treatments generally declined; the total root length of the GC group decreased to 397.5 cm, lower than that of the CK group (531.8 cm), indicating that the roots promoted by the high dose exhibited a decline in the later stage.

**Figure 9:**
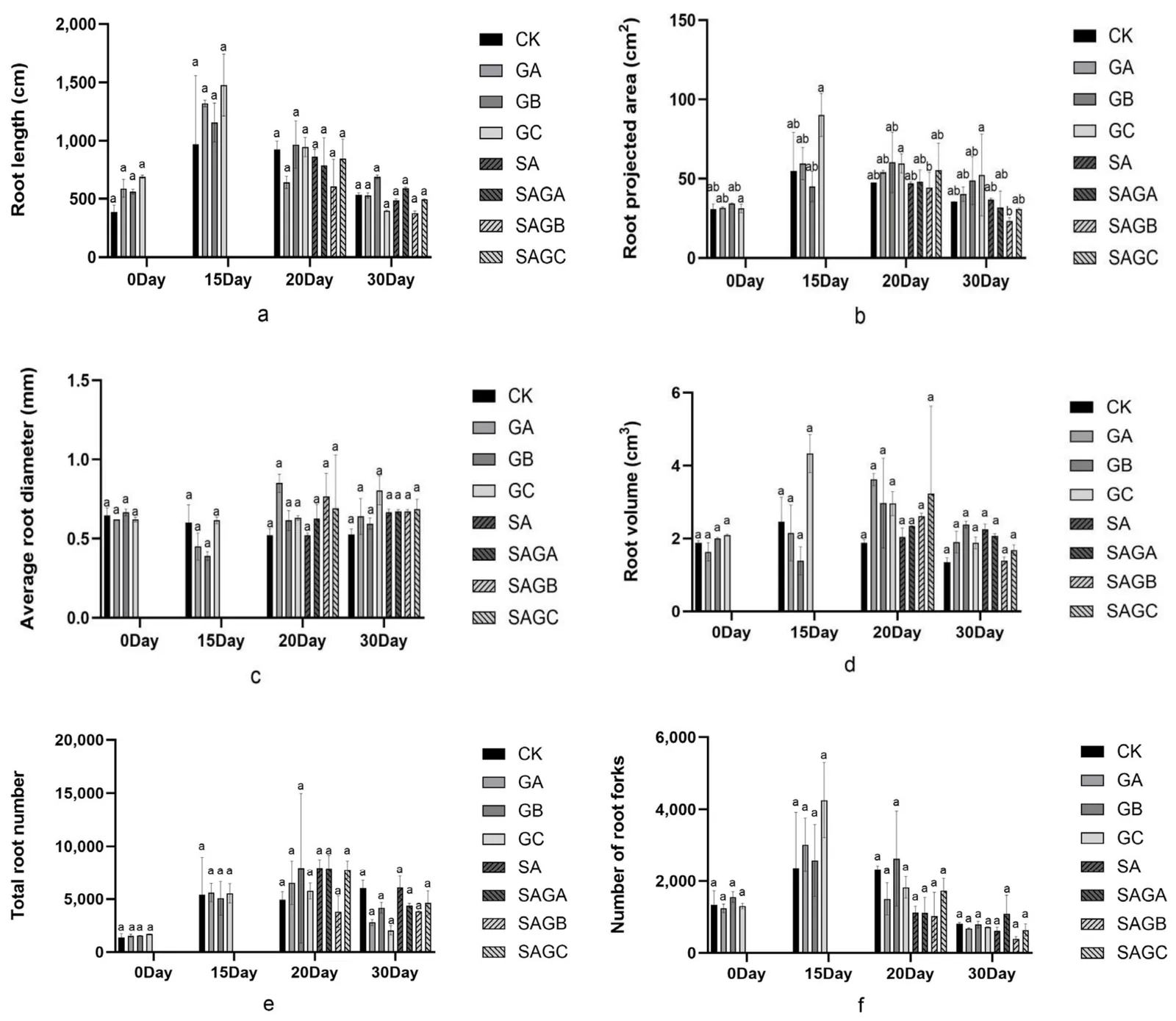
Effects of *Funneliformis mosseae* inoculation on root architectural traits of *Quercus acutissima* under 0‰ and 2‰ NaCl. Note: (**a**) total root length; (**b**) root projected area; (**c**) mean root diameter; (**d**) root volume; (**e**) total root number; (**f**) number of root forks. Treatment codes (*F. mosseae* inoculum dose, NaCl): CK (0 g, 0‰); GA (10 g, 0‰); GB (20 g, 0‰); GC (30 g, 0‰); SA (0 g, 2‰); SAGA (10 g, 2‰); SAGB (20 g, 2‰); SAGC (30 g, 2‰). Data are shown as mean ± SD (n = 3 biological replicates). Different lowercase letters indicate significant differences among treatments at each sampling time point (one-way or two-way ANOVA followed by Tukey’s multiple-comparison test, *p* < 0.05).

**Figure 10:**
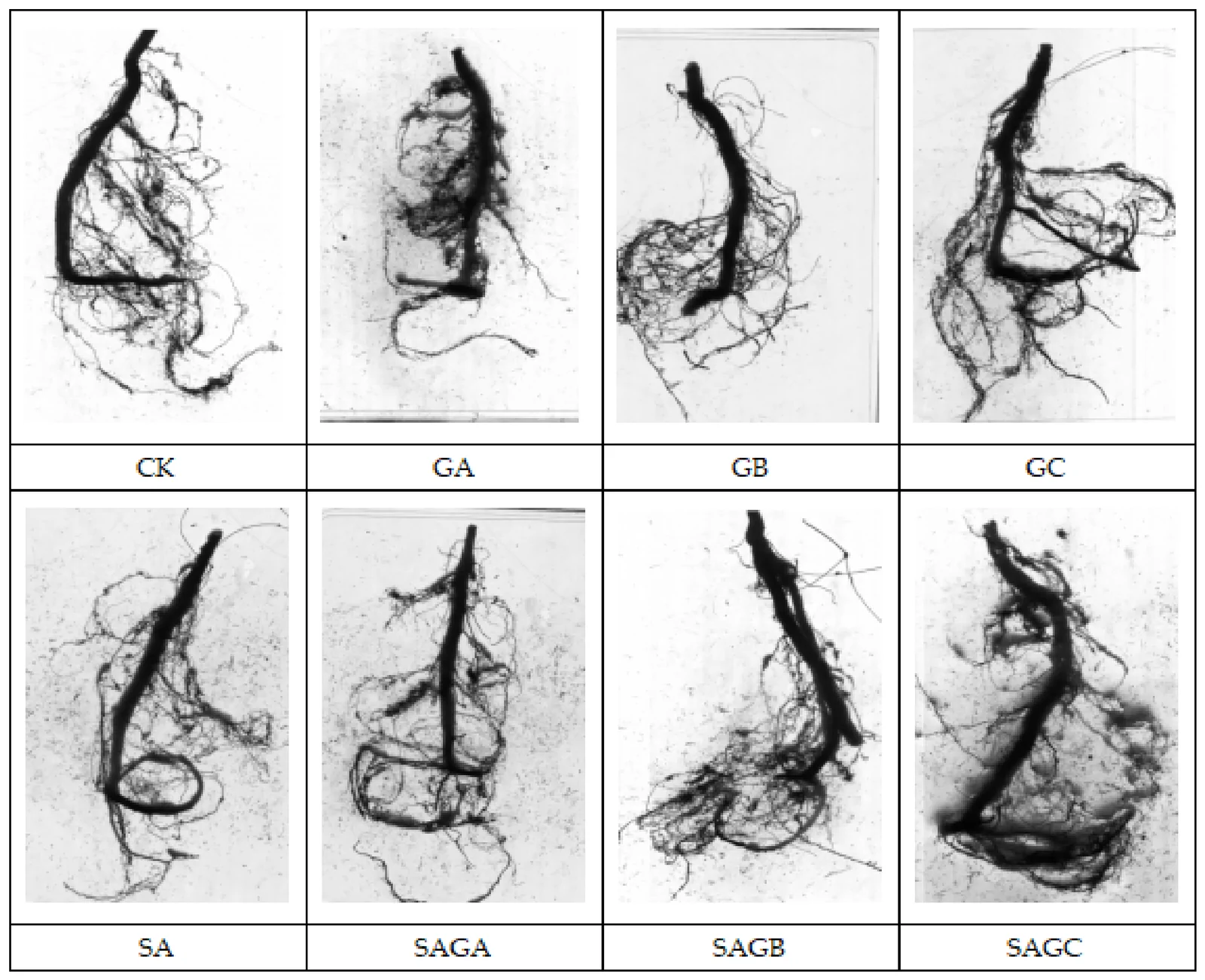
Effects of *Funneliformis mosseae* inoculation on root morphology of *Quercus acutissima* under 0‰ and 2‰ NaCl. Note: Scanned root images of each treatment group under 0‰ and 2‰ NaCl at 30 d. Treatment codes (*F. mosseae* inoculum dose, NaCl): CK (0 g, 0‰); GA (10 g, 0‰); GB (20 g, 0‰); GC (30 g, 0‰); SA (0 g, 2‰); SAGA (10 g, 2‰); SAGB (20 g, 2‰); SAGC (30 g, 2‰).

Under 2‰ NaCl conditions, salt stress inhibited root development. At 20 d, the uninoculated SA group showed a total root length of 862.1 cm, a root projected area of 47.2 cm^2^, a root volume of 2.04 cm^3^, and a number of root forks of 1117, with the total root length decreasing by 7.0% compared with the CK group (927.1 cm) under 0‰ conditions at the same time point. Inoculation partially alleviated salt injury: at 20 d, the SAGC (30 g) group showed a root projected area of 55.5 cm^2^ and a number of root forks of 1734, representing increases of 17.6% and 55.2% compared with the SA group, respectively, and a total root length of 846.6 cm, comparable to that of the SA group (862.1 cm). By 30 d, the SAGC group showed a total root length of 493.1 cm and a number of root forks of 634, still higher than those of the SA group (485.3 cm and 614, respectively), whereas the SAGB group showed a total root length of 375.9 cm and a root volume of 1.39 cm^3^, being overall weaker than the SA group (Figure 9 and Figure 10).

#### 2.3.2. SEM Observation of Root-Surface Hyphal Attachment

**Figure 11:**
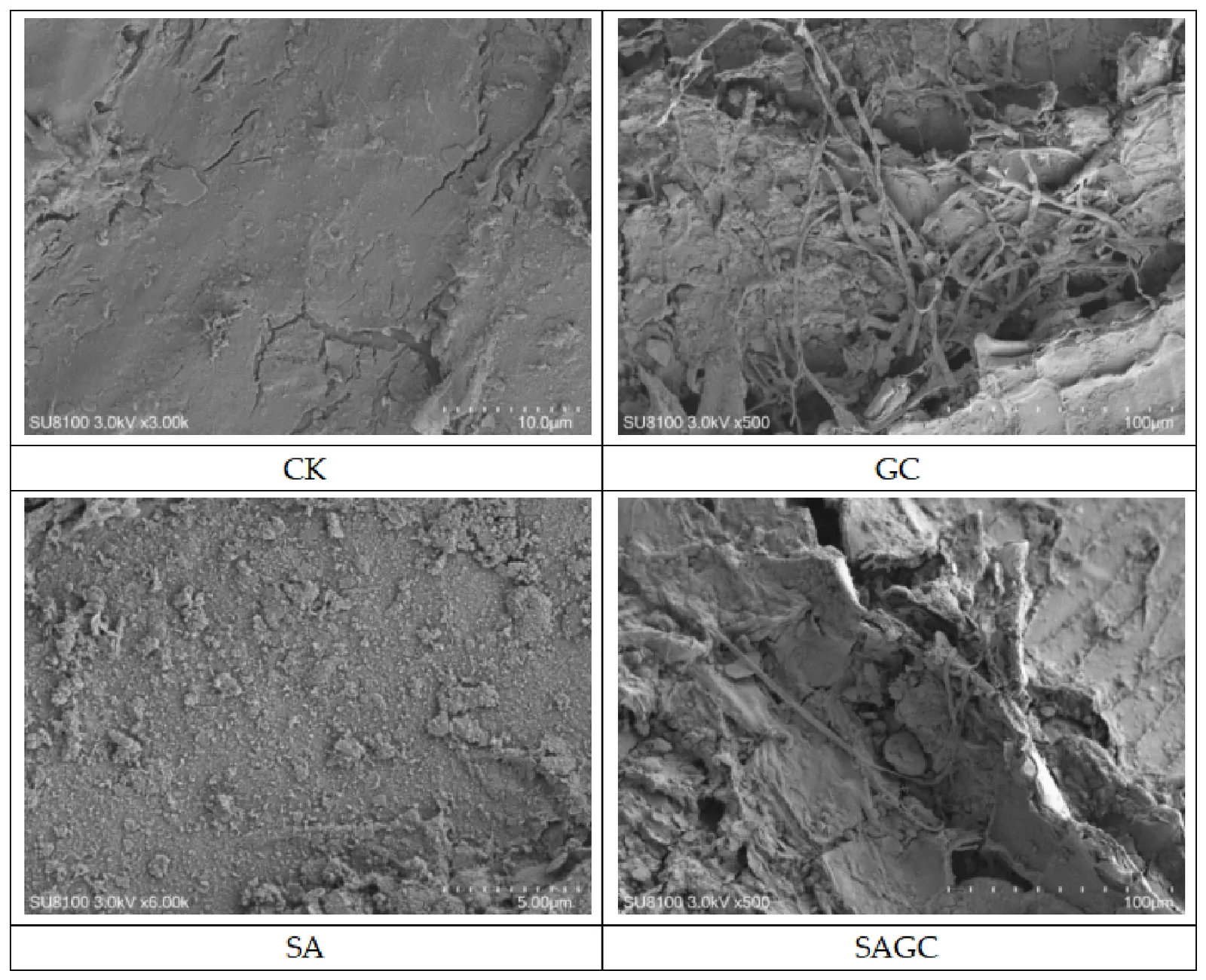
Effects of *Funneliformis mosseae* inoculation on the micromorphology of *Funneliformis mosseae* hyphae on the main root surface of *Quercus acutissima* under 2‰ NaCl. Note: attachment and morphological characteristics of *Funneliformis mosseae* hyphae on the main root surface of each treatment group at 30 d under scanning electron microscopy. CK, control group; GA, *Funneliformis mosseae*-inoculated group; SC, 2‰ NaCl salt-stressed group; SAGC, *Funneliformis mosseae*-inoculated plus 2‰ NaCl salt-stressed group. Treatment codes (*F. mosseae* inoculum dose, NaCl): CK (0 g, 0‰); GC (30 g, 0‰); SA (0 g, 2‰); SAGC (30 g, 2‰).

Scanning electron microscopy was performed on the roots of seedlings cultivated for 30 days, and obvious differences were observed in the number and morphology of *F. mosseae* hyphae on the root surface among the treatment groups. No *F. mosseae* hyphae were attached to the root surface in either the CK group or the S group. In the G and SG groups, which were the only groups inoculated with *F. mosseae*, the root surfaces were densely covered with abundant, well-developed hyphae. Under salt stress, the hyphal abundance on the roots of the SG group was significantly lower than that of the G group, and the hyphae in both inoculated groups exhibited damage such as shriveling and shrinking (Figure 11).

#### 2.3.3. Root Colonization Rate

Under 0‰ NaCl conditions, the colonization rate of the CK group remained close to 0 throughout the experiment, reaching only 0.15% at 30 d, indicating that the abundance of *F. mosseae* in the soil environment was extremely low. The colonization rates of the inoculated groups continuously increased with time: at 15 d, the colonization rates of the GB and GC groups were 8.2% and 9.1%, respectively, which increased to 20.7% and 21.5% at 30 d, representing increases of 10,250% and 10,650% compared with the CK group at the same time point, respectively. The peak colonization rate of the GB group at the late stage was slightly higher than that of the GC group, whereas the low-dose GA group showed a slower colonization rate, reaching only 11.5% at 30 d, which was 46.51% lower than that of the GC group.

**Figure 12:**
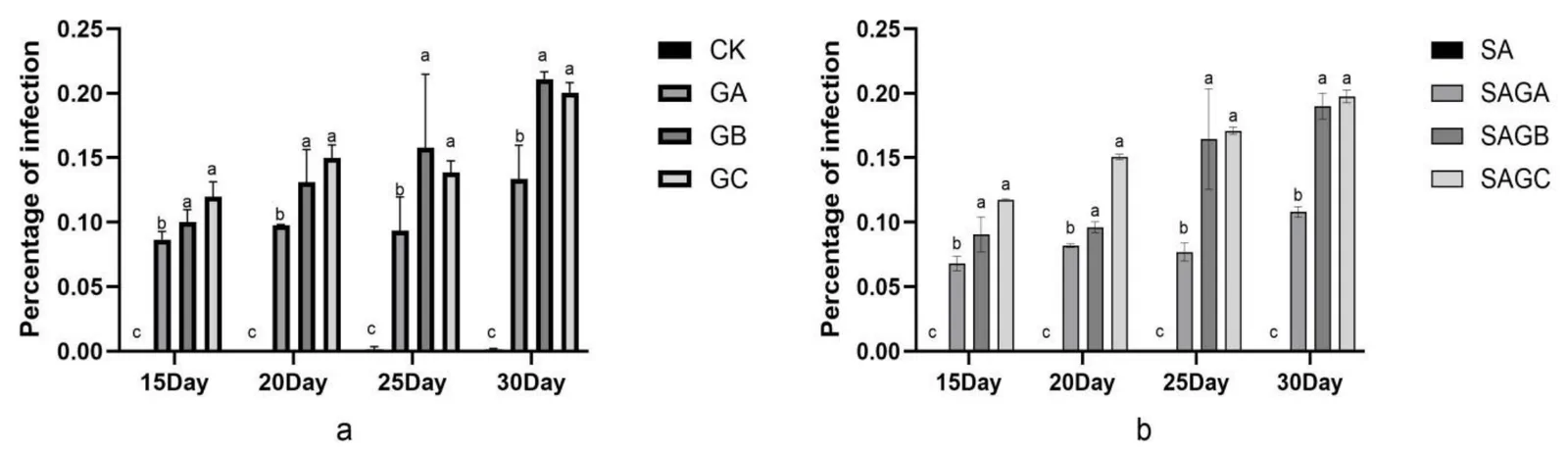
Effects of *Funneliformis mosseae* inoculation on root colonization rate of *Quercus acutissima* under 0‰ and 2‰ NaCl. Note: (**a**) *Funneliformis mosseae* colonization rate under 0‰ NaCl; (**b**) *Funneliformis mosseae* colonization rate under 2‰ NaCl. Treatment codes (*F. mosseae* inoculum dose, NaCl): CK (0 g, 0‰); GA (10 g, 0‰); GB (20 g, 0‰); GC (30 g, 0‰); SA (0 g, 2‰); SAGA (10 g, 2‰); SAGB (20 g, 2‰); SAGC (30 g, 2‰). Data are shown as mean ± SD (n = 3 biological replicates). Different lowercase letters indicate significant differences among treatments at each sampling time point (one-way or two-way ANOVA followed by Tukey’s multiple-comparison test, *p* < 0.05).

**Figure 13:**
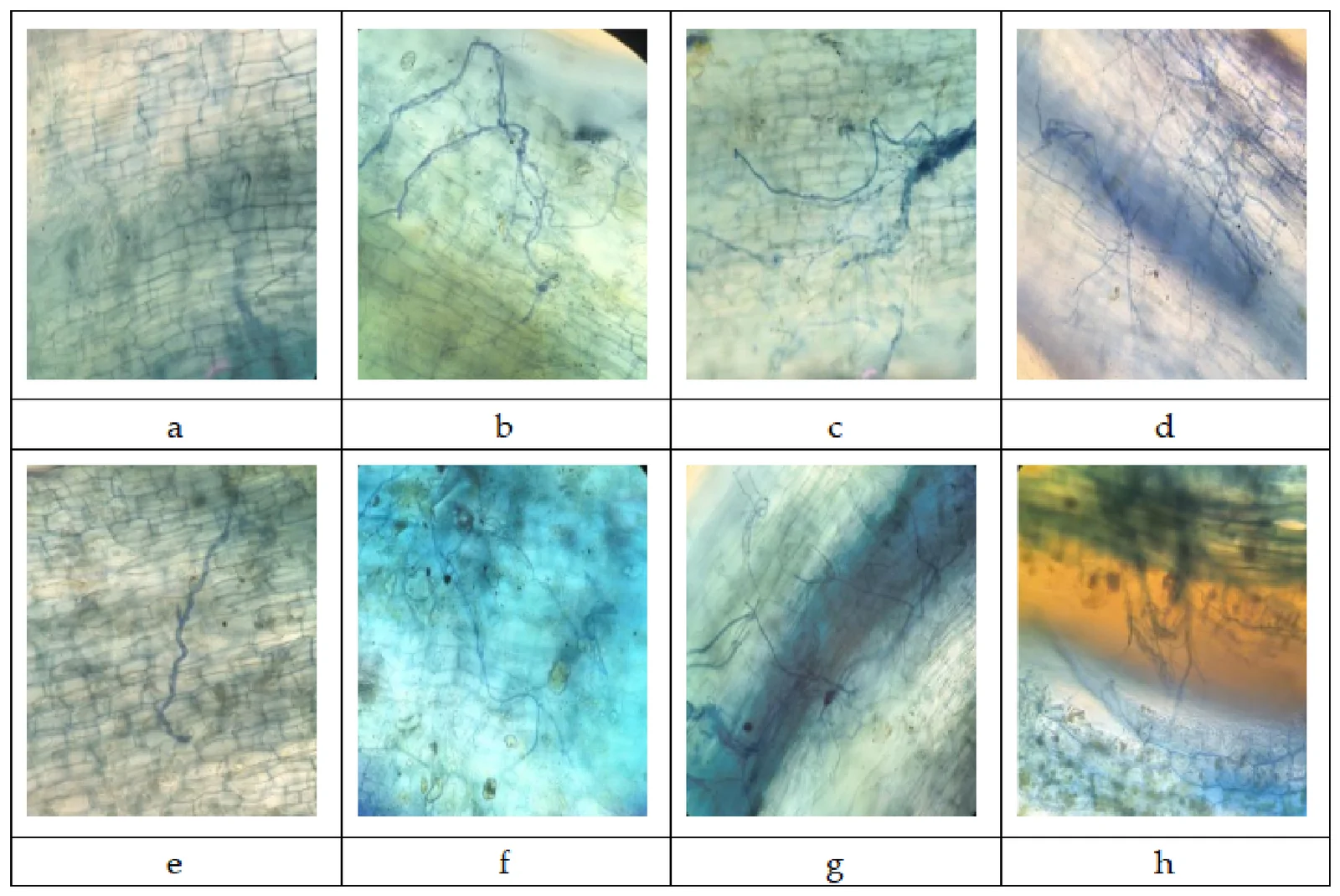
Effects of *Funneliformis mosseae* inoculation on the colonization morphology of *Funneliformis mosseae* in root cells of *Quercus acutissima under* 0‰ and 2‰ NaCl. Note: trypan blue staining showing hyphae, arbuscules and other colonization structures within root cortical cells. (**a**–**d**), treatment groups under 0‰ NaCl: (**a**) CK (uninoculated); (**b**) GA (10 g *Funneliformis mosseae*); (**c**) GB (20 g *Funneliformis mosseae*); (**d**) GC (30 g *Funneliformis mosseae*); (**e**–**h**) treatment groups under 2‰ NaCl: (**e**) SA (uninoculated); (**f**) SAGA (10 g *Funneliformis mosseae*); (**g**) SAGB (20 g *Funneliformis mosseae*); (**h**) SAGC (30 g *Funneliformis mosseae*). Scale bars for panels (**a**–**h**) = 50 μm.

Under 2‰ NaCl conditions, the colonization rate of the SA group (without exogenous inoculum) remained 0 throughout. After inoculation with *F. mosseae*, the colonization dynamics of all groups were consistent with those of the corresponding groups under 0‰ NaCl conditions, but the values were significantly lower. At 30 d, the colonization rates of the SAGB and SAGC groups were 18.3% and 19.7%, respectively, representing decreases of 11.59% and 8.37% compared with the GB and GC groups, respectively. The SAGA group exhibited the lowest colonization level, reaching only 10.5% at 30 d, which was 46.70% lower than that of the SAGC group (Figure 12 and Figure 13).

#### 2.3.4. Root Lignification and Xylem Vessel Structure

**Figure 14:**
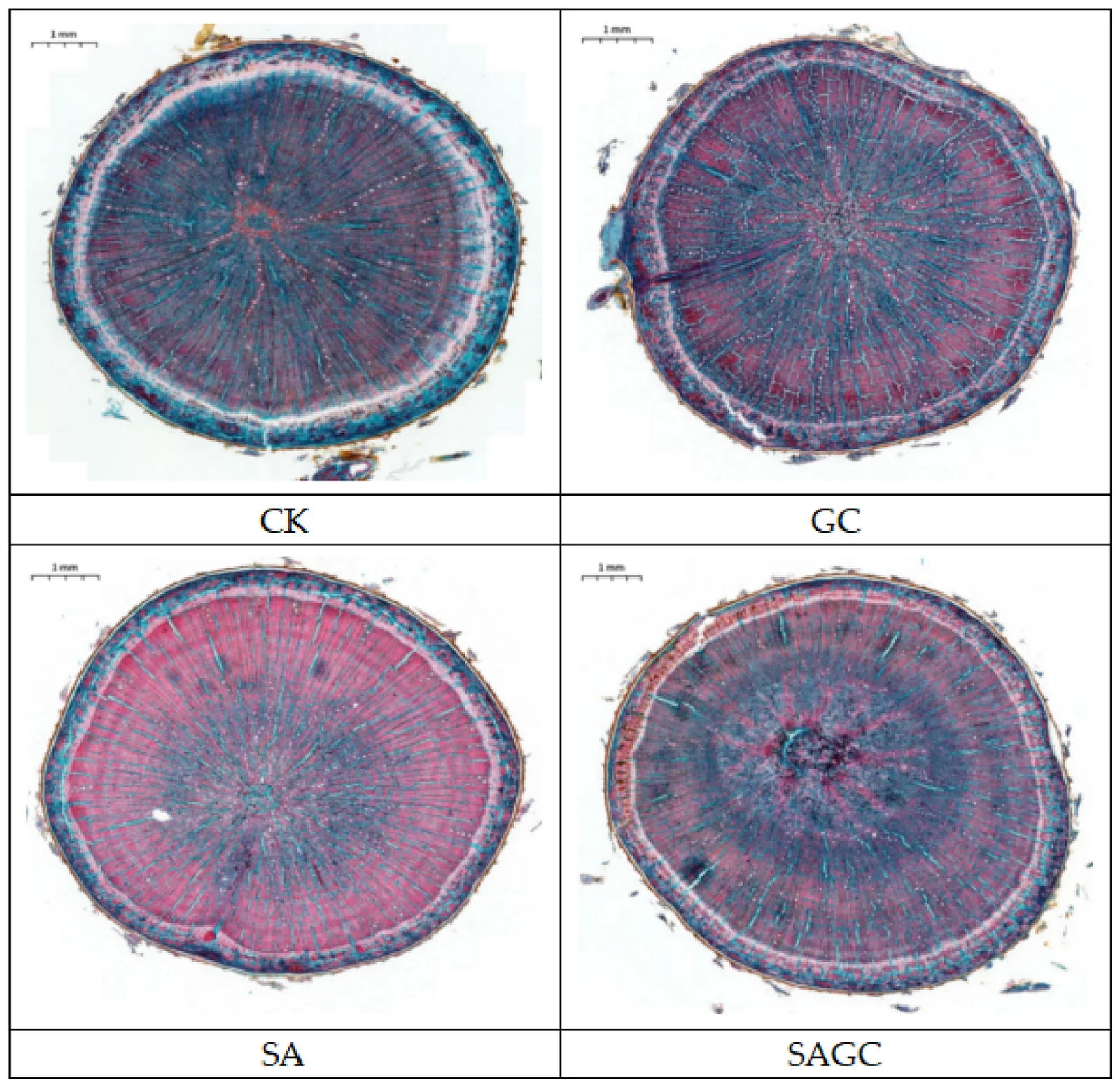
Effects of *Funneliformis mosseae* inoculation on root cross-sectional structure of *Quercus acutissima* under 2‰ NaCl. Note: safranin–fast green double staining; red staining indicates lignified tissues, and blue-green indicates cellulosic cell walls. CK, control group; GC, 30 g Funneliformis mosseae-inoculated group; SA, 2‰ NaCl salt-stressed group; SAGC, 30 g Funneliformis mosseae-inoculated plus 2‰ NaCl salt-stressed group.

**Figure 15:**
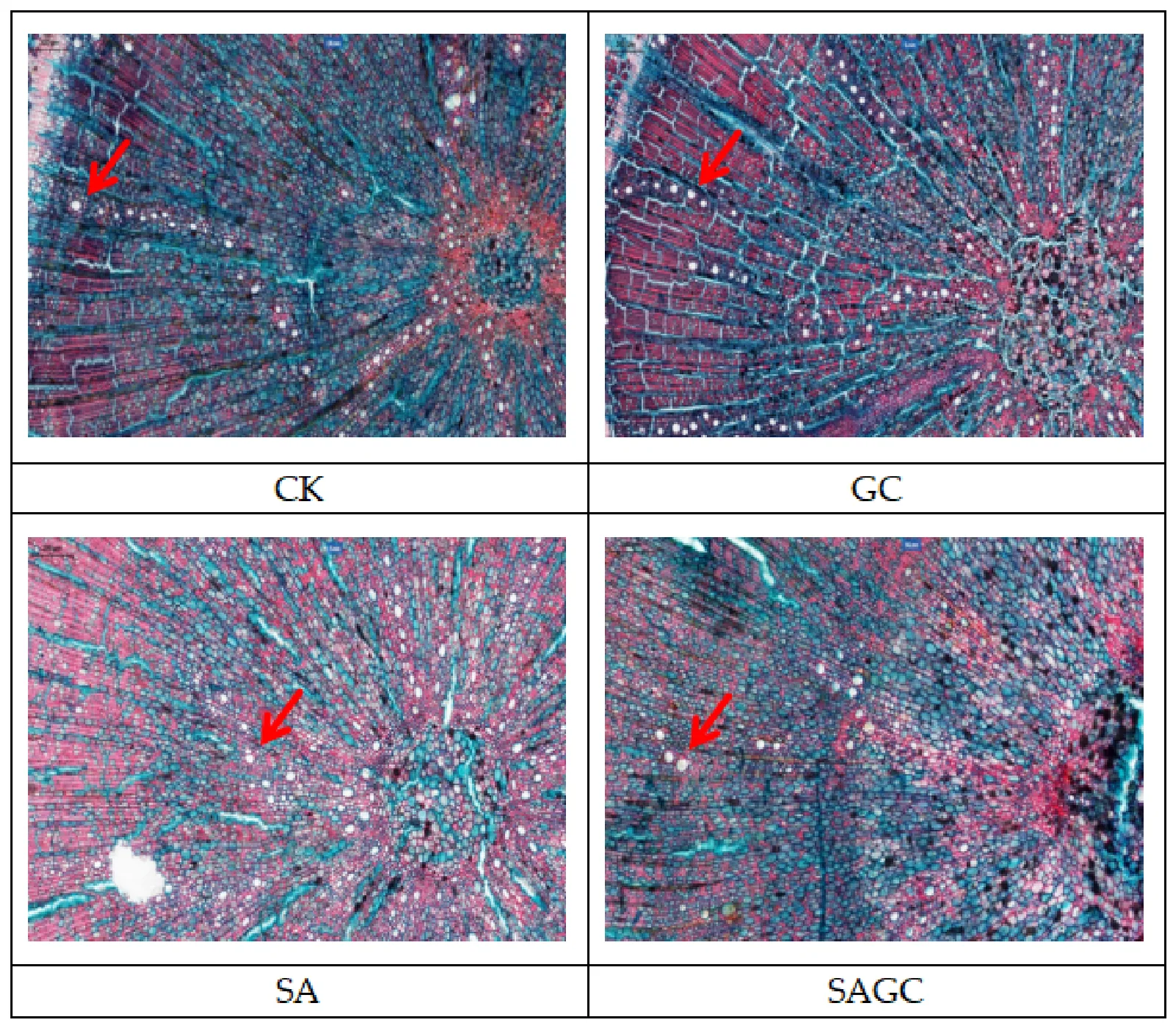
Effects of *Funneliformis mosseae* inoculation on xylem vessel morphology in root cross-sections of *Quercus acutissima* under 2‰ NaCl. Note: red arrows indicate xylem vessels. CK, control group; GC, 30 g *Funneliformis mosseae*-inoculated group; SA, 2‰ NaCl salt-stressed group; SAGC, 30 g *Funneliformis mosseae*-inoculated plus 2‰ NaCl salt-stressed group.

**Figure 16:**
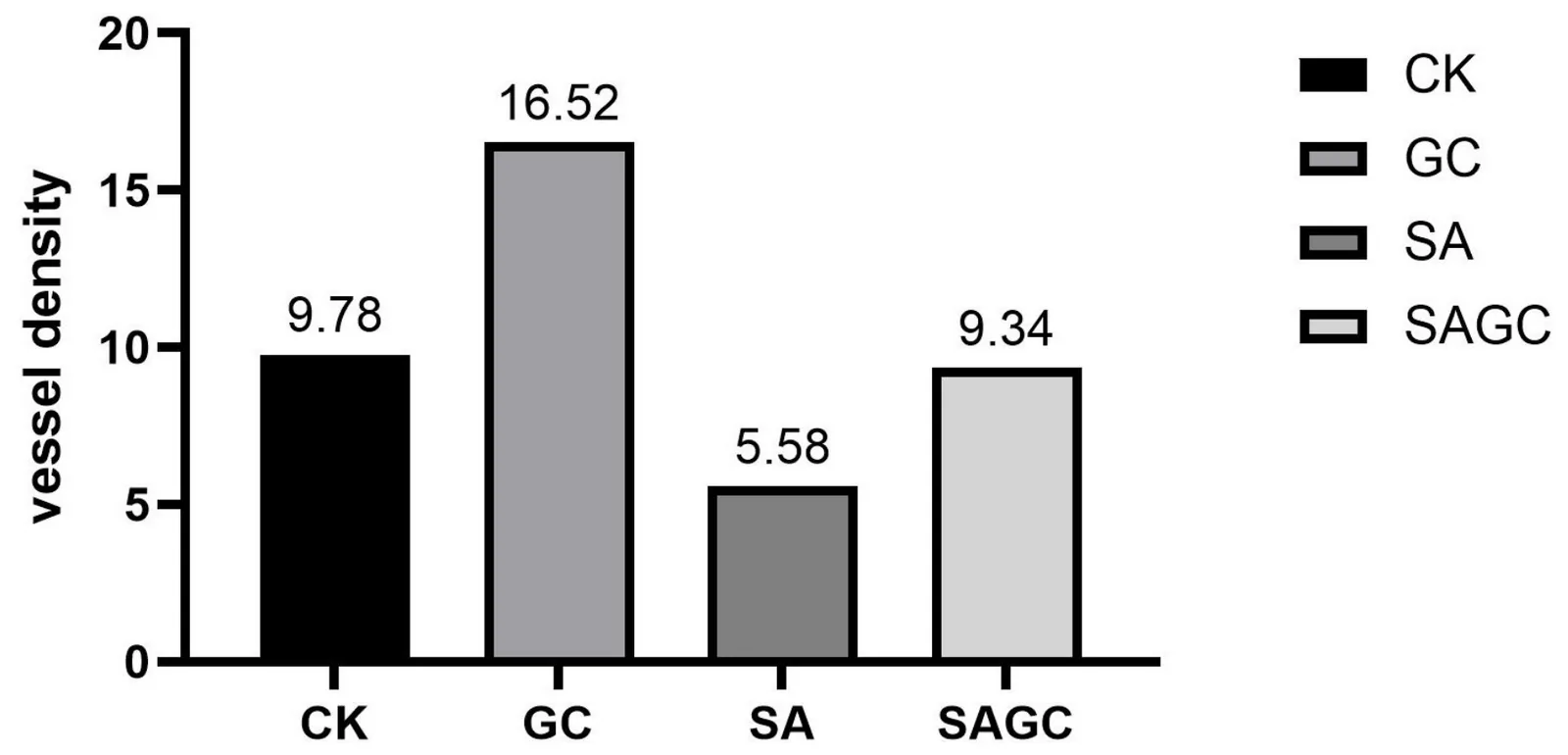
Effects of *Funneliformis mosseae* inoculation on the number of vessels per unit cross-sectional area of roots of *Quercus acutissima* under 2‰ NaCl. Treatment codes (*F. mosseae* inoculum dose, NaCl): CK (0 g, 0‰); GC (30 g, 0‰); SA (0 g, 2‰); SAGC (30 g, 2‰).

The results of safranin–fast green double staining showed that the S group exhibited the largest red-stained region, with a significantly elevated degree of lignification. The vessel density was only 5.58 vessels/mm^2^, which was 42.93% lower than that of the CK group (9.78 vessels/mm^2^), and the number of vessels per section was 107, which was 44.27% lower than that of the CK group (192). The total cross-sectional area of the S group (19.17 mm^2^) was essentially consistent with that of the CK group (19.63 mm^2^); after excluding the difference in area, the number of vessels remained significantly low, indicating that salt stress mainly inhibited vascular tissue differentiation. The vessel density of the G group increased to 16.52 vessels/mm^2^, which was 68.91% higher than that of the CK group, and its total cross-sectional area (18.34 mm^2^) was slightly lower than that of the CK group, indicating that inoculation with *F. mosseae* differentiated more vessels within a similar cross-sectional area, resulting in moderate root lignification. Lignin deposition in the SG group was significantly lower than that in the S group, and the vessel density recovered to 9.34 vessels/mm^2^, which was 67.40% higher than that of the S group and close to the level of the CK group (4.47% lower). Symbiosis with *F. mosseae* restored the suppressed vascular differentiation process (Figure 14, Figure 15 and Figure 16,Table 1).

### 2.4. Transcriptomic Mechanisms Underlying F. mosseae -Mediated Salt Tolerance

Transcriptome sequencing yielded a total of 28.89 Gb of clean data, with an average of 5.78 Gb per sample. The Q30 value exceeded 98.75% for all samples, the GC content remained stable at 44.15–44.83%, and the genome mapping rate ranged from 88.92% to 90.19%, indicating that the sequencing data quality met the required standards. Principal component analysis (PCA) revealed clear separation among the four sample groups, with reliable reproducibility within each group. A total of 31,040 expressed genes were detected. The numbers of differentially expressed genes (DEGs) in the five comparisons were 2092 (1413 upregulated and 679 downregulated), 7473 (3778 upregulated and 3695 downregulated), 6193 (3369 upregulated and 2824 downregulated), 5981 (2910 upregulated and 3071 downregulated), and 2255 (1344 upregulated and 911 downregulated), respectively. Venn diagram analysis showed that the five comparisons shared 111 core DEGs. The ML-S vs. ML-CK comparison possessed the largest number of unique genes (1416), whereas the ML-SGB vs. ML-G, ML-SGB vs. ML-CK, ML-G vs. ML-CK, and ML-SGB vs. ML-S comparisons had 677, 411, 156, and 194 unique genes, respectively. The numbers of differentially expressed transcription factors in the five comparisons were 217, 710, 614, 555, and 273, respectively. Salt stress markedly induced differential expression of transcription factors, and symbiosis with *F. mosseae* altered the overall expression scale of transcription factors under salt stress. The complete lists of differentially expressed genes for the five comparisons, together with volcano plots (Figures S1–S5), clustering heatmaps (Figures S6–S10), a Venn diagram (Figure S11), KEGG pathway enrichment analyses (Figures S12–S16), GO functional classification (Figures S17–S21), and the relative expression levels of the selected target genes verified by qRT-PCR (Figure S22), are provided in the Supplementary Materials.

GO enrichment analysis covered three categories, namely biological process (BP), cellular component (CC), and molecular function (MF). In the BP category, the core terms included oxidation–reduction, energy metabolism, salt-stress response, and hormone signaling, among which the oxidation–reduction process showed the highest enrichment in the ML-S vs. ML-CK comparison, comprising 361 genes. In the CC category, terms related to cell membrane and cell wall were significantly enriched in all comparison groups, with the plasma membrane term being most significantly enriched in the ML-S vs. ML-CK comparison, comprising 212 genes. In the MF category, terms related to oxidoreductase activity, ion transport, and transcription factor activity were mainly enriched, with ion transmembrane transporter activity being most prominently enriched in the ML-SGB vs. ML-S comparison, comprising 39 genes. A total of 127 significantly enriched pathways were identified in the KEGG analysis. Plant hormone signal transduction, pentose phosphate metabolism, inositol phosphate metabolism, and ABC transporters together constituted four core pathways in the regulatory network of *Q. acutissima* in response to salt stress and *F. mosseae* symbiosis. The hormone signaling pathway showed the highest enrichment in the ML-S vs. ML-CK comparison, with 124 enriched genes. The pentose phosphate pathway showed the highest enrichment in the ML-G vs. ML-CK comparison, with 32 enriched genes. Inositol phosphate metabolism was enriched in the ML-S vs. ML-CK comparison, with 18 enriched genes, and the ABC transporter pathway was significantly enriched in the ML-SGB vs. ML-S comparison, with 22 enriched genes.

The primer sequences of the selected key genes for qRT-PCR verification are listed in Table S1.

#### 2.4.1. Hormone Signaling Pathways Regulating Vessel Differentiation and Embolism Resistance

Combining the transcriptome gene expression data with the qRT-PCR fluorescence quantitative results, and using the blank control group (CK) as the reference, the expression patterns of the core genes (SAUR, IAA, GH3, PP2C, ERF1, and JAZ) in the four hormone signaling pathways—auxin, abscisic acid, ethylene, and jasmonic acid—were systematically analyzed under the G group (inoculation with *F. mosseae* alone), the S group (salt stress alone), and the SG group (salt stress combined with *F. mosseae* inoculation). The quantitative results effectively validated the reliability of the transcriptome sequencing data.

After inoculation with *F. mosseae* under normal growth conditions, the auxin signaling pathway was significantly induced and upregulated overall. The relative expression level of SAUR was 1.85, and those of IAA1, IAA2, and IAA3 reached 1.71, 0.08, and 7.34, respectively, with IAA3 exhibiting the most pronounced upregulation. IAA and SAUR jointly regulate vascular cell elongation, whereas only the GH3 gene was suppressed, with a relative expression level of 0.004. Similarly, in the G group, JAZ, a suppressor gene in the jasmonic acid pathway, showed a relatively high expression level. The JAZ protein can bind to MYC2 to inhibit lignin synthesis, with a relative expression level of 6.41. In contrast, the four PP2C family isoforms in the abscisic acid pathway generally maintained very low expression levels. As a negative regulator of the ABA pathway, PP2C mediates stomatal closure by regulating the SnRK2 and ABF transcription factors, thereby stabilizing the vessel water column and preventing embolism. The relative expression levels of PP2C-1, PP2C-2, PP2C-3, and PP2C-4 were 0.005, 0.264, 8.49, and 0.111, respectively. The expression of ERF1, a downstream transcription factor in the ethylene pathway, tended to be stable. ERF1 regulates the PAL and 4CL genes involved in lignin deposition, with a relative expression level of 1.11.

After exposure to salt stress, the saline adversity strongly inhibited the transcription of auxin pathway genes. The relative expression levels of SAUR, IAA1, IAA2, IAA3, and GH3 decreased to 0.10, 0.098, 0.082, 0.104, and 0.383, respectively, representing decreases of 94.59%, 94.27%, 32.50%, 98.58%, and 98.94% compared with the G group, respectively. Endogenous auxin signal transduction was blocked, and the process of cell elongation was restricted. The transcription of JAZ was significantly reduced, with the relative expression level decreasing to 0.271, a decrease of 95.77% compared with the G group. After JAZ degradation, MYC2 was released, thereby activating the jasmonic acid pathway and initiating the lignin synthesis process. Salt stress significantly activated all PP2C family genes in the abscisic acid pathway, with the relative expression levels of PP2C1–PP2C4 increasing markedly to 0.168, 4.727, 4.396, and 3.852, respectively; among them, PP2C2 showed the largest upregulation, increasing by 1690.53% compared with the G group. Abundant PP2C proteins participate in the SnRK2-ABF signaling cascade. Concurrently, the ERF1 gene was markedly upregulated to 4.19, representing an increase of 277.48% compared with the G group. The ABA and ethylene pathways cooperatively initiated the stress response, defending against osmotic injury by inducing stomatal closure and thickening of the vessel secondary wall.

**Figure 17:**
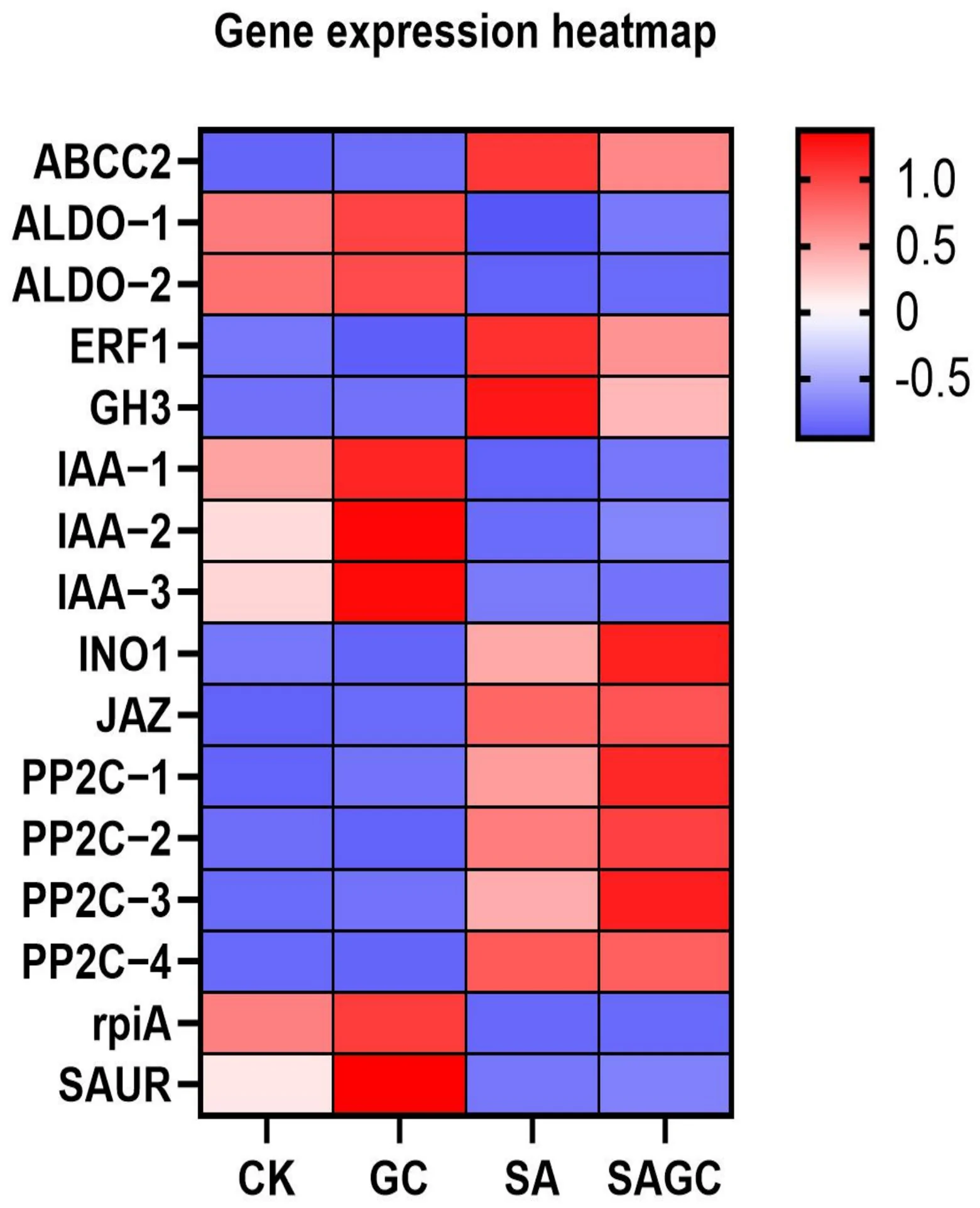
Heatmap showing the expression of selected key regulatory genes in *Quercus acutissima* after *Funneliformis mosseae* inoculation under 0‰ and 2‰ NaCl. The colour scale represents z-score-normalized gene expression values; red indicates positive (higher) expression, and blue indicates negative (lower) expression. Treatment codes (*F. mosseae* inoculum dose, NaCl): CK (0 g, 0‰); GC (30 g, 0‰); SA (0 g, 2‰); SAGC (30 g, 2‰).

**Figure 18:**
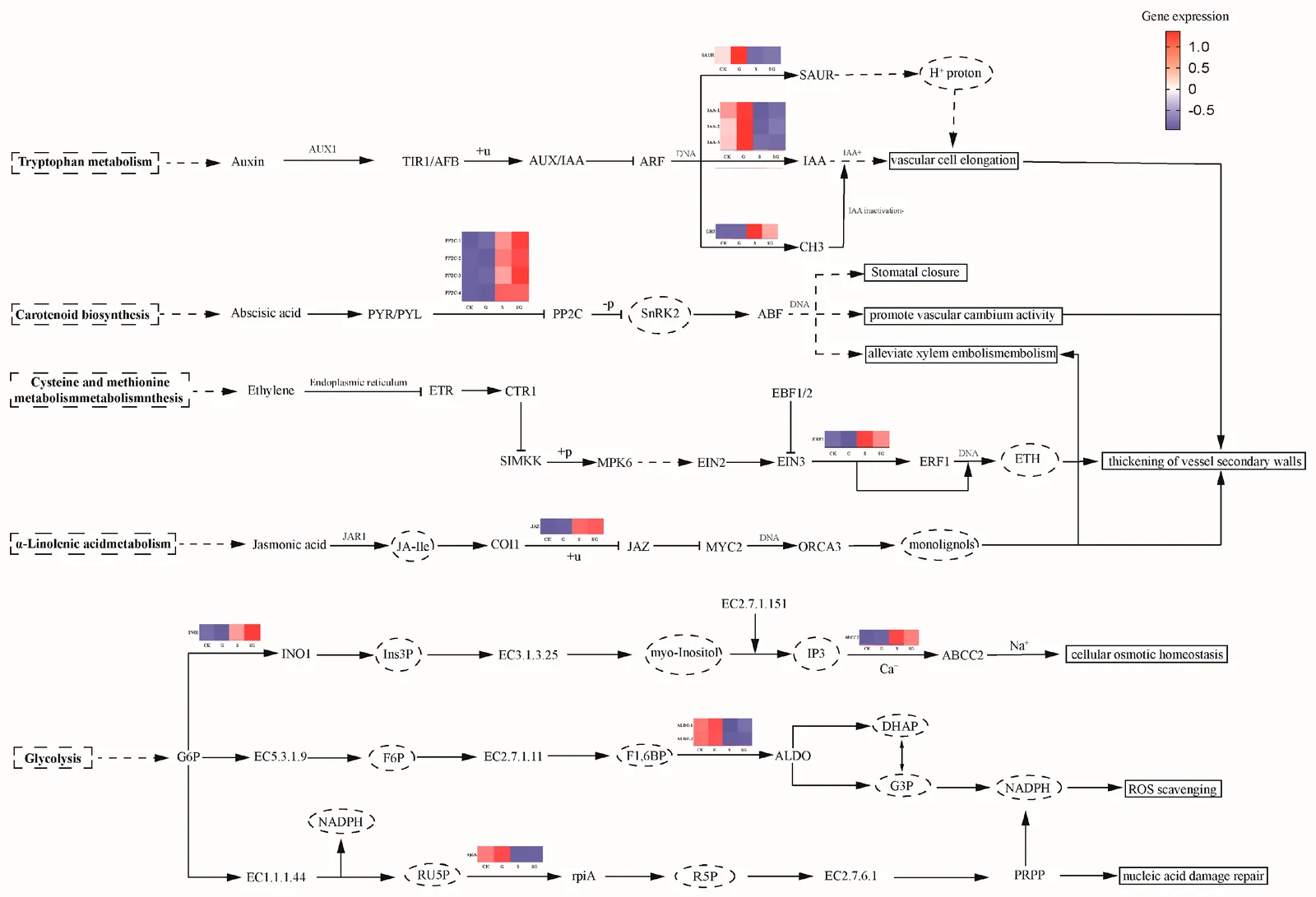
Schematic diagram of plant hormone signal transduction and carbon metabolism–Ca^2+^ signaling–ion transport pathways in *Quercus acutissima*.

After inoculation with *F. mosseae* under salt stress, the expression of auxin pathway-related genes recovered, and the expression levels of the SAUR and IAA gene families were all higher than those in the S group. The expression of JAZ also recovered concurrently, allowing moderate regulation of the jasmonic acid signaling intensity. The overall expression of PP2C family genes declined, with values falling between those of the G and S groups, continuously participating in the regulatory balance of SnRK2 and ABF. The expression level of ERF1 decreased to 1.31, representing a decrease of 68.73% compared with the S group (Figure 17 and Figure 18).

#### 2.4.2. Carbon Metabolism–Ca^2+^ Signaling–Ion Transport Pathways Regulating Redox and Ion Homeostasis

ALDO and rpiA are the genes encoding key enzymes of the pentose phosphate pathway; together they catalyze the generation of G6P and the production of NADPH, which provides reducing power for scavenging reactive oxygen species (ROS) and synthesizing proline in plants. Among the genes differentially expressed in the pentose phosphate pathway in this study, ALDO and rpiA both belong to the non-oxidative branch of this pathway, whereas the oxidative branch (e.g., glucose-6-phosphate dehydrogenase and 6-phosphogluconate dehydrogenase) was not significantly differentially expressed. INO1 is the initiating gene of inositol phosphate metabolism, which catalyzes the generation of IP3 to activate intracellular Ca^2+^ signaling. As a downstream transporter gene, ABCC2 accomplishes Na^+^ vacuolar sequestration and efflux under the drive of Ca^2+^ signaling. These three components are linked in series to constitute the carbon metabolism–Ca^2+^–ion transport regulatory axis.

In the G group, the expression levels of ALDO-1, ALDO-2, and rpiA were generally high, with relative expression levels of 0.26, 4.95, and 1.60, respectively; the ample NADPH supply ensured normal antioxidant and osmotic regulation in the plants. INO1 and ABCC2 maintained relatively low expression levels of 0.84 and 0.11, respectively; in the absence of salt ion toxicity, there was no need to extensively initiate the ion transport program.

The S group significantly altered the expression of genes in the three pathways. The relative expression levels of ALDO-1, ALDO-2, and rpiA decreased to 0.04, 0.73, and 0.19, respectively, representing decreases of 84.62%, 85.25%, and 88.13% compared with the G group, respectively. The pentose phosphate pathway was weakened, NADPH synthesis decreased, and the basal antioxidant capacity of the plants declined. The expression of INO1 and ABCC2 was markedly upregulated, reaching 5.06 and 4.24, respectively, representing increases of 502.38% and 3754.55% compared with the G group, respectively. The abundant INO1 synthesized IP3 to activate Ca^2+^ signaling, which in turn induced ABCC2 to synthesize large amounts of transporters, accelerating the sequestration of excess intracellular Na^+^ and alleviating salt ion toxicity.

The pathway diagram results for the SG group showed that the expression of the three pentose phosphate pathway genes ALDO-1, ALDO-2, and rpiA generally recovered compared with the S group. The expression of INO1 increased compared with the S group, with a relative expression level of 0.39, whereas that of ABCC2 markedly declined, with a relative expression level of 0.06, representing a decrease of 98.58% compared with the S group (Figure 17 and Figure 18).

## 3. Discussion

### 3.1. Regulatory Patterns of F. mosseae Inoculation and Salt Stress on the Growth, Root Architecture, and Anatomical Structure of Q. acutissima Seedlings

Under 0‰ NaCl conditions, the GB and GC groups, relying on intact mycorrhizal symbiotic structures, widened the range of nitrogen and phosphorus nutrient uptake from the soil; photosynthates were preferentially allocated to the stems and newly emerged leaves, and root meristematic activity was continuously activated, resulting in growth rates of plant height, ground diameter, and various root indicators significantly superior to those of the CK group. Under 2‰ NaCl conditions, free sodium ions damaged the root meristematic tissue of the SA group, directly slowing the growth of plant height and ground diameter and reducing the overall root size. In contrast, the mycorrhizal symbiosis of the SAGB and SAGC groups blocked excess salt ions from contacting the root epidermis and widened the range of water and nutrient uptake; their growth rates of plant height, ground diameter, leaf number, and various root indicators were all superior to those of the SA group, with the 30 g inoculation exhibiting a better growth-promoting effect than the 20 g.

The data showed that the two habitats (0‰ and 2‰ NaCl) exhibited a consistent pattern: *Q. acutissima* inoculated with 20 g or 30 g of *F. mosseae* promoted the growth of plant height, ground diameter, leaf number, total root length, root volume, total root number, and number of root forks, with the growth-promoting effect increasing with increasing inoculation dose. In contrast, Q. acutissima seedlings inoculated with 10 g of *F. mosseae* failed to form a fully functional symbiotic system; although it promoted plant height under both 0‰ and 2‰ NaCl, it did not consistently improve ground diameter or physiological indicators and even aggravated their inhibition under salt stress [16].

### 3.2. Response Characteristics of F. mosseae Root Colonization and Hyphal Attachment Under Salt Stress

Combining the root colonization rate data with the scanning electron microscopy (SEM) observations, it was evident that the *F. mosseae* hyphae attached to the root surface exhibited shriveled and shrunken morphology regardless of salt treatment. Compared with the 0‰ NaCl cultivation condition, the root colonization rate of *F. mosseae* was generally lower after salt treatment, and the number of fungal hyphae attached to the main root surface was markedly reduced. Free Na^+^ in the soil disrupts the nutrient uptake and proliferation processes of *F. mosseae* hyphae, restricts the continued colonization of hyphae on the root epidermis, and directly reduces the overall root colonization level [17,18]. Meanwhile, in *Q. acutissima* plants without exogenous *F. mosseae* inoculum, neither root sections nor SEM observations detected obvious colonization structures of *F. mosseae*, and no traces of hyphal attachment were found on the root surface, reflecting the extremely low content of *F. mosseae* in the soil used in this experiment; only artificial supplementation with exogenous inoculum could establish a stable mycorrhizal symbiosis [19].

### 3.3. Osmotic Regulation Mechanism of F. mosseae Inoculation in Q. acutissima Under Salt Stress

Combining the gene expression characteristics of the pentose phosphate pathway and the inositol phosphate-mediated Ca^2+^ signaling pathway, it was evident that inoculation with *F. mosseae* under salt-free conditions upregulated key enzyme-encoding genes of the pentose phosphate pathway, such as ALDO and rpiA. The enhanced metabolic efficiency of the pathway increased NADPH synthesis, simultaneously activating the synthesis of endogenous antioxidant and osmotic regulatory substances, and NADPH also participates in the synthesis of chlorophyll a [20]. The measurement data showed that, after inoculation with *F. mosseae*, the total chlorophyll content of the seedlings increased markedly, and the accumulation levels of POD, PRO, MDA, and EC were all higher than those of the CK group. The stress-related substances at the endogenous metabolic level increased synchronously, and *Q. acutissima* exhibited stress-like response characteristics at the physiological level.

Under salt stress, the expression of ALDO and rpiA was downregulated, and the insufficient NADPH supply directly limited the synthesis of antioxidant enzymes. The SA group, which was not inoculated with *F. mosseae*, lacked mycorrhiza-mediated pathway regulation; consequently, POD activity rapidly declined after reaching its peak, chlorophyll a underwent substantial degradation owing to the lack of reducing-power support, and the overall chlorophyll content decreased accordingly. In the SAGB and SAGC groups, the increasing inoculation dose attenuated the downregulation of ALDO and *rpiA*; the steadily produced NADPH not only supported the long-term function of POD but also alleviated the degradation of chlorophyll a, and the decline rate of POD after its peak gradually slowed with increasing *F. mosseae* inoculation. The NADPH generated by the pentose phosphate pathway can provide reducing power for membrane lipid antioxidant reactions, directly regulating the generation and accumulation of MDA. In the SA group, with low expression of ALDO and rpi*A* and insufficient NADPH, membrane lipid peroxidation progressed continuously, and the MDA content increased rapidly with stress duration and fluctuated markedly. After inoculation with *F. mosseae*, ALDO and rpiA maintained relatively stable expression levels to provide sufficient reducing power for scavenging cellular reactive oxygen species and suppressing the chain reaction of membrane lipid peroxidation. Consequently, the overall MDA content of the inoculated seedlings changed gently; mycorrhizal symbiosis effectively alleviated cell membrane oxidative damage, the chloroplast membrane structure was protected, and the total chlorophyll content did not decline substantially [21].

The inositol phosphate pathway gene INO1 maintained an upregulated expression state in the SA group, catalyzing the abundant generation of IP3 to continuously activate intracellular Ca^2+^ signaling and inducing the sustained and substantial synthesis of the osmotic regulatory substance proline (PRO). Accordingly, in the SA group, the PRO content continued to increase with stress duration. Symbiosis with *F. mosseae* downregulated the expression intensity of *INO1*, reducing IP3 generation, moderating the Ca^2+^ signaling intensity, and stabilizing the PRO synthesis rate. In the SAGC group inoculated with 30 g, the INO1 expression further decreased from day 25 to day 30, accompanied by reduced PRO synthesis and a declining trend in content. The expression level of ABCC2, a downstream transporter gene of Ca^2+^ signaling, directly regulates the efficiency of intracellular Na^+^ sequestration and efflux. In the SA group, the continuous IP3 stimulation upregulated ABCC2 expression; the intracellular ion imbalance induced drastic changes in cell membrane permeability, resulting in markedly elevated and fluctuating EC values. Inoculation with *F. mosseae* weakened the INO1–IP3–Ca^2+^ signaling cascade, allowing ABCC2 to maintain a moderate expression level and thereby maintaining intracellular Na^+^ homeostasis. As a result, the overall EC values of all *F. mosseae* -inoculated treatment groups were lower than those of the SA group, with markedly reduced fluctuation [22].

The NADPH steadily produced by the pentose phosphate pathway continuously ensures POD catalytic activity, retards the excessive accumulation of MDA, and alleviates the degradation of chlorophyll a. The inositol phosphate–Ca^2+^ signaling pathway balances the PRO synthesis rate and intracellular ion distribution by regulating the expression of INO1 and ABCC2. The two pathways cooperatively regulate the dynamic changes in five physiological indicators, namely POD, MDA, PRO, EC, and chlorophyll, jointly alleviating the physiological damage caused by salt stress to *Q. acutissima* seedlings from the multiple dimensions of antioxidant defense, osmotic regulation, and ion homeostasis [23].

It should be noted that increased pentose phosphate pathway activity cannot be inferred solely from transcript abundance. Although the upregulation of the non-oxidative-branch genes ALDO and rpiA was consistent with enhanced carbon flux supporting NADPH regeneration and redox homeostasis, direct biochemical evidence—such as enzyme activity assays and the NADPH/NADP^+^ ratio—is required to confirm enhanced pathway activity and NADPH synthesis. Such biochemical validation will be incorporated in future studies.

### 3.4. Mechanism by Which F. mosseae Inoculation Enhances Salt Tolerance in Q. acutissima by Alleviating Root Xylem Conductive Embolism

IAA, SAUR, and GH3 in the auxin pathway, together with PP2C in the abscisic acid pathway, jointly participate in the regulation of xylem vessel cell elongation and expansion; JAZ in the jasmonic acid pathway, in coordination with ERF1 in the ethylene pathway, is responsible for the accumulation of lignin in the vessel secondary wall. These six core genes collectively determine the final morphology of the root xylem.

After inoculation with *F. mosseae* under salt-free conditions, the expression levels of IAA and SAUR increased, whereas that of GH3 was suppressed; the processes of vessel cell division and elongation proceeded continuously, and the vessel density reached 16.52 vessels/mm^2^, which was 68.91% higher than that of the CK group (9.78 vessels/mm^2^), indicating a markedly increased vessel density. Mycorrhizal symbiosis also induced the upregulation of PP2C and the simultaneous upregulation of JAZ, weakening the lignin synthesis effect mediated by jasmonic acid signaling; consequently, the red-stained region of the root sections was larger and the degree of lignification was higher than that of the blank control. The changes in root anatomical structure corresponded to the physiological data, indicating that inoculation with *F. mosseae* under normal growth conditions induced a slight stress-like response in *Q. acutissima*.

Under salt stress, the expression of IAA and SAUR was significantly suppressed, whereas that of GH3 was elevated; the vessel differentiation and expansion processes were impeded, and the vessel density decreased to 5.58 vessels/mm^2^, which was 42.93% lower than that of the CK group (9.78 vessels/mm^2^), with a marked reduction in newly formed vessels. Salt stress also suppressed the expression of JAZ, releasing MYC2 to activate the lignin synthesis pathway; together with the sustained upregulation of PP2C and ERF1, the red coverage of the root sections increased substantially. The excessive accumulation of lignin caused over-lignification of the roots, narrowing the vessel lumens, aggravating xylem embolism, and reducing water transport capacity [24]. Under salt stress, inoculation with *F. mosseae* re-elevated the expression of IAA and SAUR and suppressed that of GH3, restoring the vessel differentiation capacity; the vessel density recovered to 9.34 vessels/mm^2^, which was 67.40% higher than that of the salt-stressed group and close to the level of the CK group. Meanwhile, the expression of JAZ was upregulated and those of PP2C and ERF were downregulated, suppressing lignin synthesis metabolism [25]. The red-stained region of the sections shrank, the degree of root lignification decreased, vessel blockage was alleviated, and xylem embolism damage was effectively relieved.

The alterations in root and vessel anatomical traits are closely linked to salt tolerance. High salinity restricts vessel differentiation and increases the risk of xylem embolism, which reduces water transport efficiency and aggravates ion accumulation in root tissues. Inoculation with *F. mosseae* alleviated these anatomical disorders, maintained relatively stable vessel structure, and thereby guaranteed water and nutrient transport under salt stress. Such anatomical modulation is an important morphological strategy for Q. acutissima to cope with saline environments.

It should be noted that the paraffin section observation of root anatomy in the present study was conducted only at a single sampling time point (day 30 after salt treatment). Since salt-induced root lignification and xylem embolism are dynamic and progressive processes, this single-endpoint sampling cannot fully capture the continuous developmental changes in root lignification and vessel differentiation under salt stress combined with mycorrhizal symbiosis; consequently, the anatomical data presented here represent a static snapshot of the responses at day 30. In future studies, time-course anatomical observations at intermediate time points will be performed to more precisely resolve the dynamic progression of root lignification and vessel differentiation, and to further verify the alleviating effect of mycorrhizal symbiosis on xylem dysfunction under saline conditions.

From a practical standpoint, these findings have direct implications for nursery inoculation and field deployment in coastal shelterbelts. Because the 30 g high-dose inoculant was the most effective and the beneficial effect of mycorrhizal symbiosis was confined to mild salinity (≤2‰), we recommend inoculating Q. acutissima seedlings with a sufficient *F. mosseae* dose (e.g., 20–30 g per seedling) at the nursery stage to allow mycorrhizal establishment before outplanting, and deploying such mycorrhizal seedlings mainly in coastal saline areas where the soil salt content does not exceed approximately 2‰. As demonstrated for soybean on saline–alkaline lands [26], translating molecular understanding into field-level salt tolerance requires integrating genotype selection, optimized inoculant application, and agronomic management; the dose–response and salinity-threshold relationships established here provide a practical reference for such integrated deployment.

## 4. Materials and Methods

### 4.1. Study Site

The experimental site is located at the Forestry Experiment Station of Shandong Agricultural University, Tai’an City, Shandong Province, China, and is characterized by a warm-temperate continental semi-humid monsoon climate, with a multi-year mean annual temperature of 12.9 °C, an annual mean precipitation of approximately 697 mm, an annual mean relative humidity of about 65%, an annual sunshine duration of 2400–2600 h, and a frost-free period of approximately 195 days.

### 4.2. Plant Materials and Fungal Inoculum

In this study, one-year-old half-sib family seedlings of *Q. acutissima* were used as experimental materials. The seed-source mother trees were superior individuals located in Weihai City, Shandong Province. Seeds were collected in September 2024, and plump seeds of uniform size were selected and sown in the autumn of the same year. In July 2025, seedlings with consistent growth were selected for the subsequent pot experiment.

The inoculation doses (10, 20, and 30 g) refer to the fresh weight of the soil-based inoculant product rather than to purified fungal material. Based on the spore density of 20 spores g^−1^, the 10, 20, and 30 g doses corresponded to approximately 200, 400, and 600 spores per seedling, respectively. To exclude substrate interference, uninoculated control treatments received equal-weight autoclaved inoculum substrate.

### 4.3. Experimental Design

A two-factor completely randomized block design was adopted, with four NaCl salinity gradients (0‰, 2‰, 4‰, and 6‰) and four *F. mosseae* inoculation dose gradients (0, 10, 20, and 30 g inoculant). The two factors were fully crossed to constitute a full factorial design with a total of 16 treatment combinations; accordingly, an uninoculated control (0 g inoculant) was present at every salinity level (0‰, 2‰, 4‰, and 6‰). To exclude interference from the carrier substrate and other inoculum components, all uninoculated control treatments received equal-weight autoclaved inoculum substrate (Section 4.2). This full factorial design allowed the independent effects of salinity, inoculation, and their interaction to be differentiated. Each treatment combination included three biological replicates, and each replicate consisted of five *Q. acutissima* seedlings(Table 2).

After the seedlings had been inoculated with *F. mosseae* and cultivated for 15 days, gradient salt-stress treatments were applied. Saline solutions were prepared by adding 0, 2, 4, and 6 g of sodium chloride (NaCl) to every 1000 g of water to obtain the corresponding concentrations. Herein, NaCl concentrations are expressed in per mille (‰), denoting grams of NaCl per kilogram of water (mass fraction, w/w); accordingly, the 2‰, 4‰, and 6‰ treatments correspond to 0.2%, 0.4%, and 0.6% (w/w) NaCl, respectively. The solutions were then applied to individual seedlings in small amounts on multiple occasions until the soil salinity reached the designated level. During the experiment, the seedlings were watered on an as-needed basis (i.e., whenever the soil surface became dry) using a mixture of the corresponding salt solution and fresh water. After each watering, the soil electrical conductivity (EC_1:5_, 1:5 soil–water extract) was measured with a soil conductivity meter and converted to the corresponding salt concentration, thereby ensuring that the target salinity was maintained in each treatment group throughout the experiment. The corresponding saturated paste electrical conductivity (Ece) and soil salt content (Tse) for each salinity gradient are presented in Table 3.

### 4.4. Data Collection and Processing

#### 4.4.1. Determination of Growth and Morphological Traits

Aboveground growth indicators: plant height was determined as the vertical distance from the root collar junction to the terminal bud and measured with a steel ruler with an accuracy of 1 mm; ground diameter was measured with a vernier caliper with an accuracy of 0.01 mm at 2 cm above the ground surface, and the mean of measurements in the east–west and north–south perpendicular directions was calculated; and the number of leaves was counted based on fully expanded functional leaves. All of the above indicators were measured and recorded for each seedling at 0, 15, 20, 25, and 30 days after the start of the experiment.

Belowground root architectural indicators: the whole plants were harvested, and the root surfaces were thoroughly rinsed with clean water. All roots were collected through a 0.5 mm sieve and scanned with an Epson Perfection V850 Pro (Seiko Epson Corporation, Suwa, Nagano, Japan) root scanner at a resolution of 400 dpi. The scanned images were processed using WinRHIZO Pro 2019 (Regent Instruments Inc., Quebec, Canada) root analysis software to obtain six parameters, namely total root length, root projected area, root volume, mean root diameter, total root number, and number of root forks, which were measured and recorded for each seedling at 0, 15, 20, and 30 days after the start of the experiment.

#### 4.4.2. Determination of Leaf Physiological Indicators

POD activity was determined by the guaiacol colorimetric method, and the enzyme activity was calculated from the absorbance measured at 470 nm using an ultraviolet (UV) spectrophotometer [27]; MDA content was determined by the thiobarbituric acid (TBA) colorimetric method, and the content was converted from the absorbance values measured at 532, 600, and 450 nm [28]; Proline (Pro) content was calculated from the absorbance measured at 520 nm after color development with acidic ninhydrin in a boiling water bath [29]; chlorophyll was extracted with 80% acetone in the dark, and the absorbance was measured at 663 and 645 nm to calculate the contents of chlorophyll a, chlorophyll b, and total chlorophyll [30]; relative leaf electrical conductivity was determined by soaking equal amounts of fresh leaves in deionized water, measuring the initial conductivity after standing at room temperature, and measuring the final conductivity after boiling in a boiling water bath; and the relative conductivity was then calculated from the ratio of the two values [31].

#### 4.4.3. Determination of *F. mosseae* Colonization Rate

Fresh lateral roots were collected every 5 days starting from day 15 of the experiment and cut into 1 cm segments. The segments were macerated in 10% KOH at 90 °C for 30 min, acidified with 2% HCl for 5 min, stained with 0.05% trypan blue in lactoglycerol at 90 °C for 30 min, and destained in lactoglycerol. Thirty root segments were randomly examined per sample under a microscope, and the root colonization rate was calculated as (number of colonized segments/total number of segments) × 100% [32].

#### 4.4.4. Observation of Micromorphological Characteristics of the Main Root Surface

At day 30 of the experiment, fresh main roots were collected from each treatment group, cut into approximately 0.5 cm segments, and fixed in 2.5% glutaraldehyde at 4 °C for 24 h. After rinsing three times with 0.1 mol/L phosphate buffer (pH 7.2), the segments were dehydrated through a graded ethanol series (30–100%), subjected to tert-butanol replacement drying, and sputter-coated with gold (approximately 15 nm thick). The samples were then observed under a SU8010 scanning electron microscope (Hitachi, Japan) to examine the distribution, attachment, and micromorphological characteristics of *F. mosseae* hyphae on the root surface [33].

#### 4.4.5. Observation of the Anatomical Structure of the Main Root

At day 30 of the experiment, fresh main roots were collected from each treatment group, cut into approximately 0.5 cm segments, and fixed in FAA fixative (formalin:glacial acetic acid:70% ethanol = 5:5:90) for 24 h. The segments were then dehydrated through a graded ethanol series, cleared in xylene, embedded in paraffin, and sectioned at a thickness of 8 μm using a rotary microtome. The sections were floated and mounted on glass slides in warm water at 42 °C. After drying, the sections were dewaxed in xylene, rehydrated through a graded ethanol series, stained with 1% safranin for 2 h, counterstained with 0.5% fast green for 30 s, dehydrated, cleared, and mounted in neutral balsam. The degree of root lignification and the morphology of vascular tissues were observed under an Olympus BX53 (Olympus Corporation, Tokyo, Japan) light microscope, and the number of vessels was counted in five randomly selected 10 × 10 fields of view per sample [34].

#### 4.4.6. Transcriptome Sequencing and Bioinformatics Analysis

Functional leaf tissues were collected at day 30 from four representative treatment groups—CK (uninoculated, 0‰ NaCl), G (inoculated with *F. mosseae*, 0‰ NaCl), S (uninoculated, 2‰ NaCl), and SG (inoculated with *F. mosseae*, 2‰ NaCl)—and total RNA was extracted from three biological replicates per group. After quality assessment (RIN ≥ 7.0), sequencing libraries were constructed for transcriptome sequencing. Using the *Q. acutissima* reference genome (accession number: GWHBGBO00000000), which is publicly available from the National Genomics Data Center (NGDC, https://ngdc.cncb.ac.cn/gwh/(accessed on 21 March 2026)), differentially expressed genes were identified (log_2_FC ≥ 1, q < 0.05), and GO and KEGG enrichment analyses were performed. Key genes, including SAUR, IAA, PP2C, JAZ, ERF1, ALDO, rpiA, INO1, and ABCC2, were selected and validated by SYBR-based quantitative real-time PCR (qRT-PCR) using GAPDH as the internal reference, and the relative expression levels were calculated by the 2^−ΔΔCt^ method [35]. The qRT-PCR validation results are shown in Figure S1 (relative expression levels of the selected genes), and the primer sequences are listed in Table S1.

#### 4.4.7. Data Processing

All measured data were subjected to one-way and two-way analysis of variance (ANOVA) using DPS7.05 statistical software, and multiple comparisons among treatment groups were performed using Tukey’s test. Each treatment contained three biological replicates (n = 3) at each sampling time point, with five seedlings included in each biological replicate; the measured values of the five seedlings within each biological replicate were averaged to yield a single value per replicate. Statistical analysis was performed independently for each sampling time point. Different lowercase letters in figures indicate significant differences among treatments at *p* < 0.05. *p* < 0.05 was considered statistically significant, and *p* < 0.01 was considered highly significant. In cases where apparent differences among mean values were not statistically significant, this was attributed to high within-group biological variability and the limited number of biological replicates (n = 3), which reduced the statistical power of Tukey’s test. Data visualization was performed using GraphPad software.

## 5. Conclusions

This study demonstrated that, under salt-free conditions, *Quercus acutissima* inoculated with any dose of *Funneliformis mosseae* exhibited a slight stress-like response, with abnormal accumulation of antioxidant and osmotic-regulatory physiological substances. Regarding the salt-stress adaptation threshold, inoculation with *Funneliformis mosseae* effectively enhanced the salt tolerance of *Quercus acutissima* under mild salt conditions of 2‰ NaCl or below; however, under high-salt conditions of 4‰ NaCl or above, which exceeded the tolerance threshold of the plant–mycorrhizal symbiosis system, none of the *F*unneliformis mosseae doses could alleviate salt injury, and all seedlings eventually died. In terms of the inoculation dose effect, the 10 g low-dose inoculum failed to form a fully functional mycorrhizal symbiosis and exerted inconsistent effects: it promoted plant height under both 0‰ and 2‰ NaCl but showed no consistent improvement in ground diameter or physiological indicators and even aggravated their inhibition under salt stress; in contrast, the 30 g high-dose inoculum exhibited the optimal effect in enhancing salt tolerance. It improved the water transport structure by increasing the vessel density and alleviating root over-lignification and regulated multiple stress-resistance physiological indicators by enhancing POD activity, reducing MDA and PRO accumulation, maintaining cell membrane stability, and slowing chlorophyll degradation. At the transcriptional level, the pentose phosphate pathway, the inositol phosphate–Ca^2+^ signaling pathway, and the plant hormone pathways coordinately regulated antioxidant defense, osmotic balance, and root lignification, collectively achieving the salt tolerance conferred by mycorrhizal symbiosis.

## Figures and Tables

**Table 1:** Statistics of vessel number per unit cross-sectional area of *Quercus acutissima* roots inoculated with AMF under different NaCl concentrations.

Group	Total Vessel Number	Total Cross-Sectional Area (mm^2^)	Vessel Number per Unit Area (Vessels/mm^2^)
CK	192	19.63	9.78
G	303	18.34	16.52
S	107	19.17	5.58
SG	168	17.98	9.34

**Table 2:** Designation and naming rules of treatment groups.

NaCl Concentration	*Funneliformis mosseae* Inoculation Dose			
	0 g	10 g	20 g	30 g
0‰	CK	GA	GB	GC
2‰	SA	SAGA	SAGB	SAGC
4‰	SB	SBGA	SBGB	SBGC
6‰	SC	SCGA	SCGB	SCGC

**Table 3:** Soil electrical conductivity and salt content under different NaCl treatments.

NaCl Mass Concentration	EC_1:5_ (dS/m)	Ece (dS/m)	Tse (‰)	Tse (g/kg)
0 ‰	0.43	0.97	0.62	0.62
2 ‰	1.49	3.14	2.01	2.01
4 ‰	3.00	6.22	3.98	3.98
6 ‰	4.57	9.42	6.03	6.03

Note: Ece (dS/m) = 2.0428 × EC1:5 + 0.0895; Tse (g/kg) = 0.64 × Ece = 1.307392 × EC1:5 + 0.05728. EC1:5, electrical conductivity of the 1:5 soil–water extract (dS/m); Ece, saturated paste electrical conductivity (dS/m); Tse, soil salt content, expressed in per mille (‰) or grams per kilogram (g/kg); because 1‰ = 1 g/kg, the values of Tse (‰) and Tse (g/kg) are numerically equal.

## Data Availability

The original contributions presented in this study are included in the article. Further inquiries can be directed to the corresponding authors.

## Supplementary Materials

The following supporting information can be downloaded at: https://www.mdpi.com/article/10.3390/plants15213450/s1: Figure S1, Relative expression levels of selected target genes; Figure S2, Heatmap of relative expression levels of selected target genes; Figure S3 Volcano plot of differentially expressed genes between ML-S and ML-CK; Figure S4 Volcano plot of differentially expressed genes between ML-G and ML-CK; Figure S5 Volcano plot of differentially expressed genes between ML-SGB and ML-CK; Figure S6 Volcano plot of differentially expressed genes between ML-SGB and ML-G; Figure S7 Volcano plot of differentially expressed genes between ML-SGB and ML-S; Figure S8 Cluster heatmap of differentially expressed genes between ML-S and ML-CK; Figure S9 Cluster heatmap of differentially expressed genes between ML-G and ML-CK; Figure S10 Cluster heatmap of differentially expressed genes between ML-SGB and ML-CK; Figure S11 Cluster heatmap of differentially expressed genes between ML-SGB and ML-G; Figure S12 Cluster heatmap of differentially expressed genes between ML-SGB and ML-S; Figure S13 Venn diagram of differentially expressed genes; Figure S14 KEGG pathway enrichment analysis between ML-S and ML-CK; Figure S15 KEGG pathway enrichment analysis between ML-G and ML-CK; Figure S16 KEGG pathway enrichment analysis between ML-SGB and ML-CK; Figure S17 KEGG pathway enrichment analysis between ML-SGB and ML-G; Figure S18 KEGG pathway enrichment analysis between ML-SGB and ML-S; Figure S19 GO functional classification between ML-S and ML-CK; Figure S20 GO functional classification between ML-G and ML-CK; Figure S21 GO functional classification between ML-SGB and ML-CK; Figure S22 GO functional classification between ML-SGB and ML-G; Figure S23 GO functional classification between ML-G and ML-SGB; Table S1, Primer sequences used for qRT-PCR analysis in *Quercus acutissima*.
