# Supplementary material for: Physiological and Transcriptional Mechanisms Underlying Salt Tolerance in Quercus acutissima Regulated by Funneliformis mosseae
Source: Plants (Basel). 2026 Nov 5;15(21):3450. doi: 10.3390/plants15213450 (PMC13766008; doi:10.3390/plants15213450)
Supplement: Supplementary file 1 [file plants-15-03450-s001.zip › plants-4687184-supplementary.pdf]

Supplementary Figures

1.Gene Expression

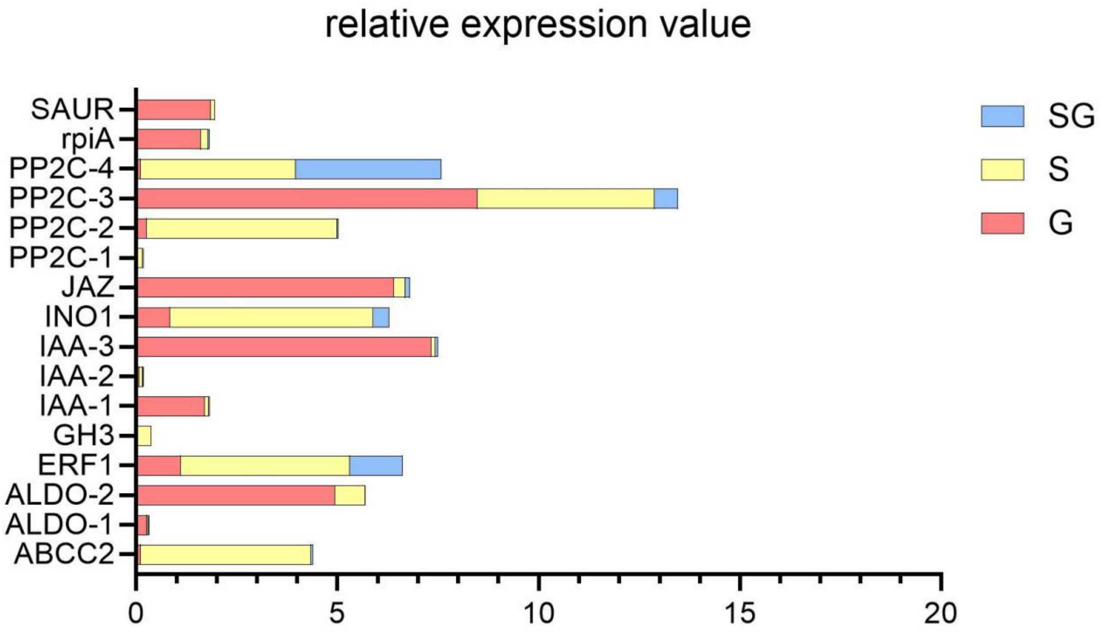

Figure S1 Relative expression levels of selected target genes

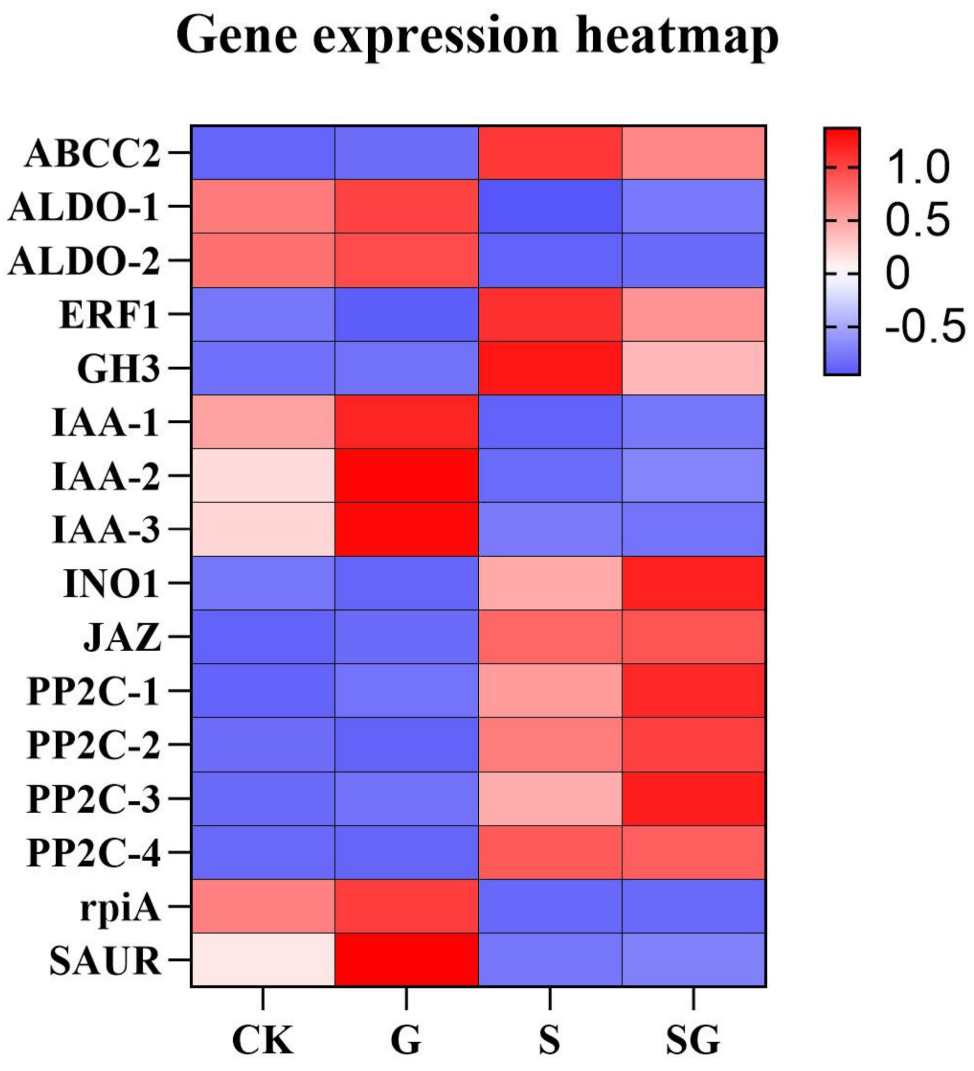

Figure S2 Heatmap of relative expression levels of selected target genes

2.Volcano plots of differentially expressed genes

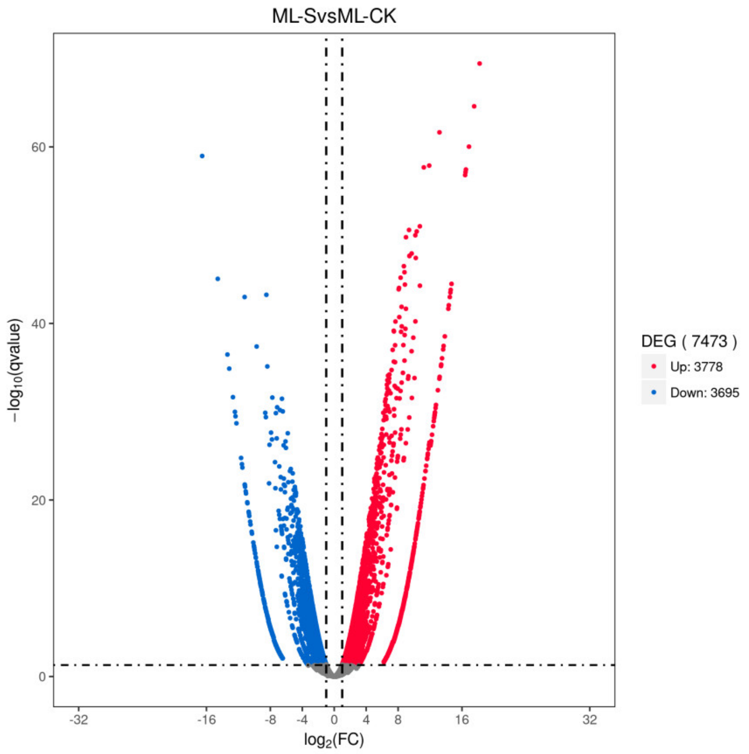

Figure S3 Volcano plot of differentially expressed genes between ML-S and ML-CK

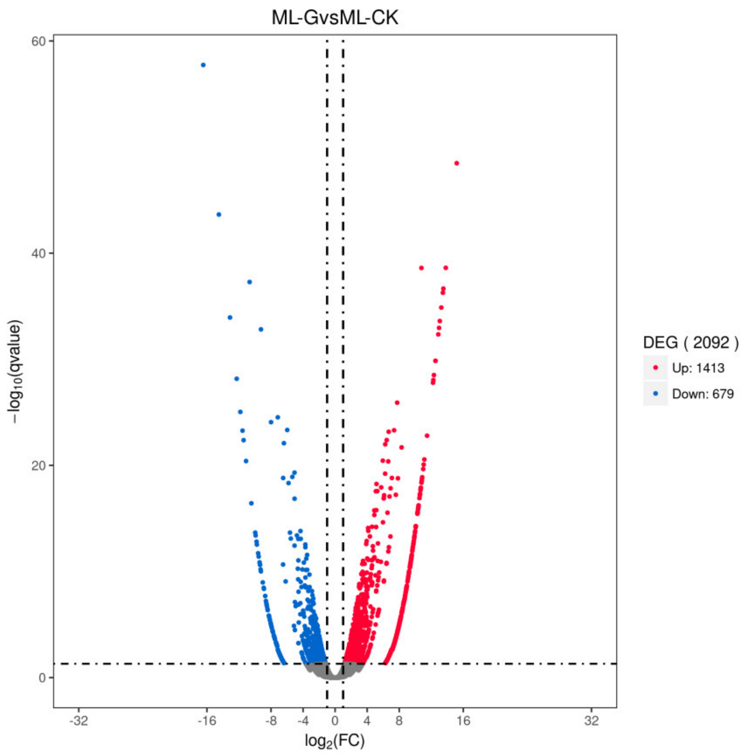

Figure S4 Volcano plot of differentially expressed genes between ML-G and ML-CK

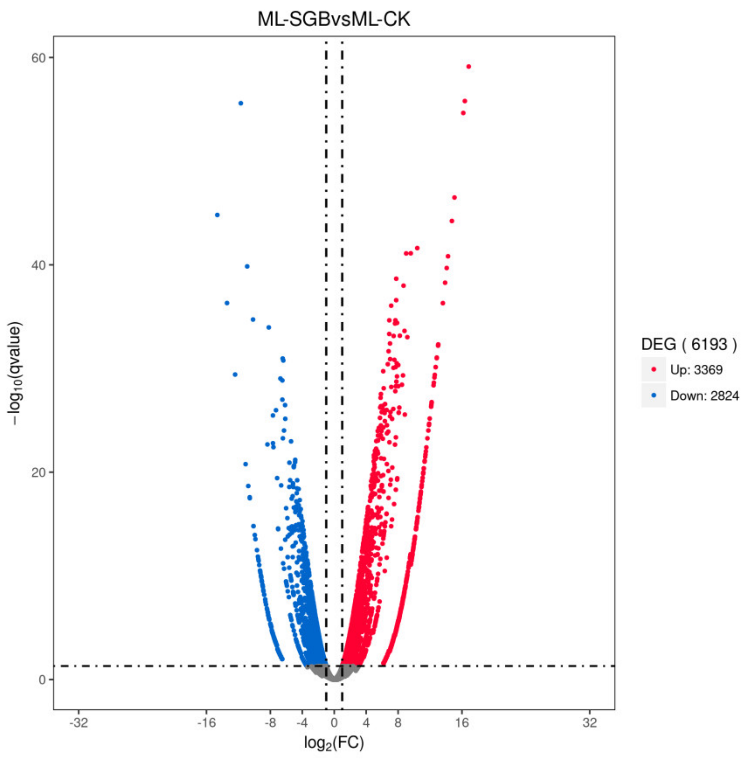

Figure S5 Volcano plot of differentially expressed genes between ML-SGB and ML-CK

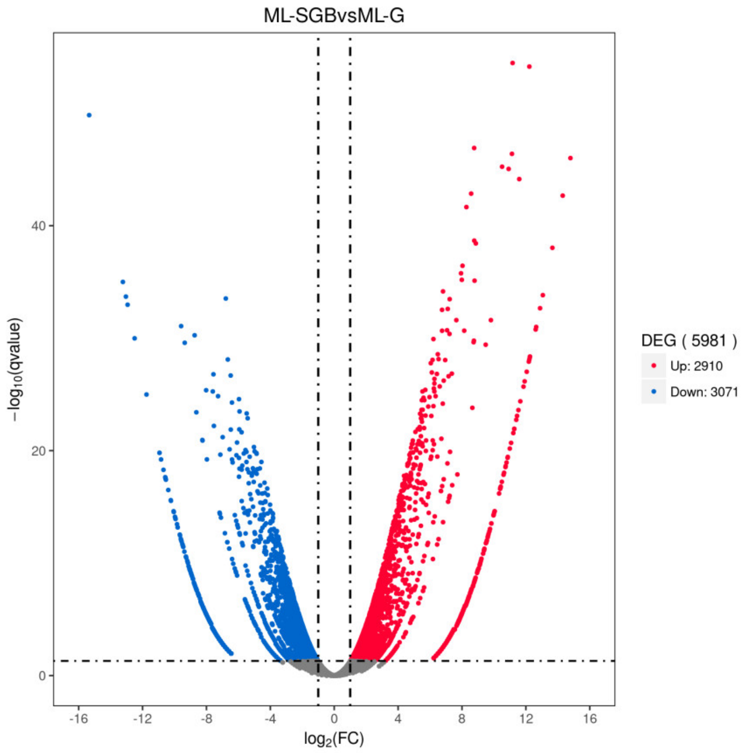

Figure S6 Volcano plot of differentially expressed genes between ML-SGB and ML-G

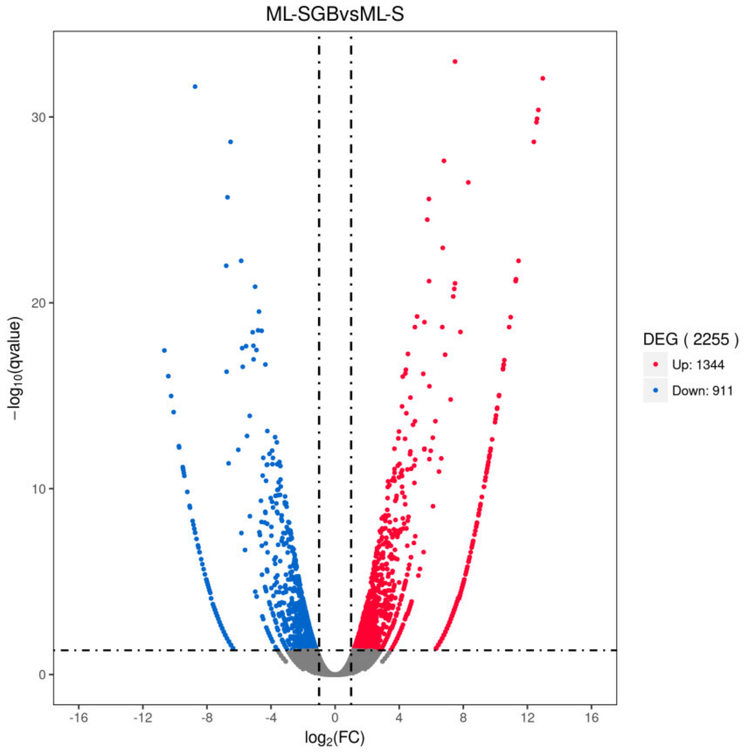

Figure S7 Volcano plot of differentially expressed genes between ML-SGB and ML-S

3.Heatmaps of Differentially Expressed Genes

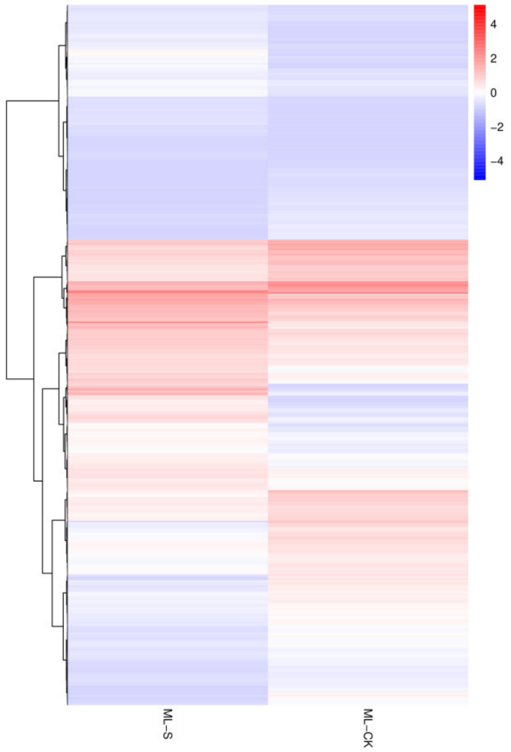

Figure S8 Cluster heatmap of differentially expressed genes between ML-S and ML-CK

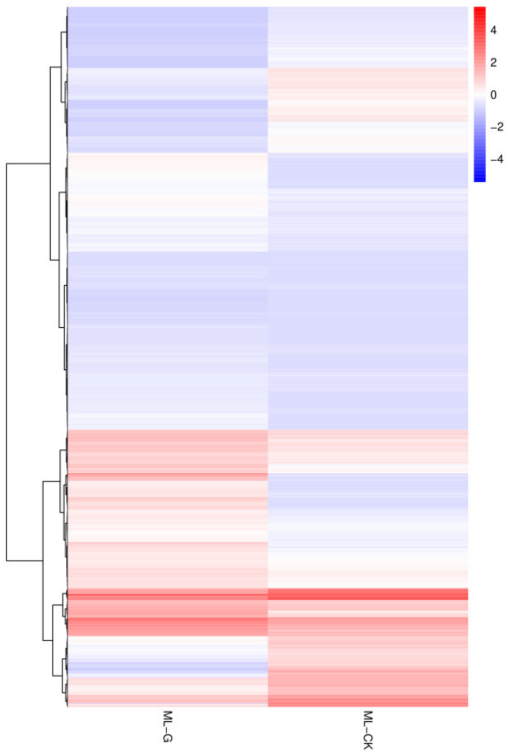

Figure S9 Cluster heatmap of differentially expressed genes between ML-G and ML-CK

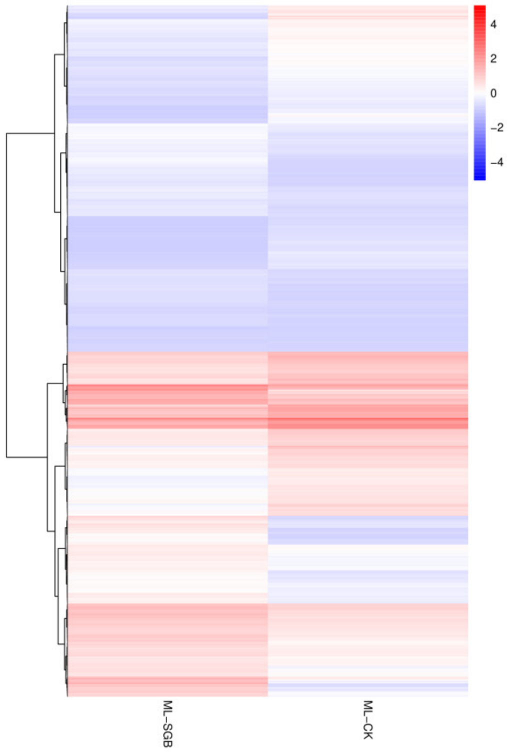

Figure S10 Cluster heatmap of differentially expressed genes between ML-SGB and ML-CK

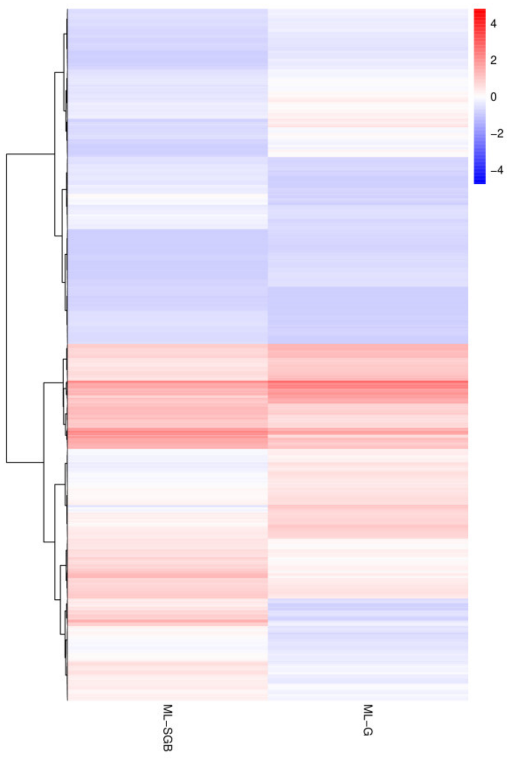

Figure S11 Cluster heatmap of differentially expressed genes between ML-SGB and ML-G

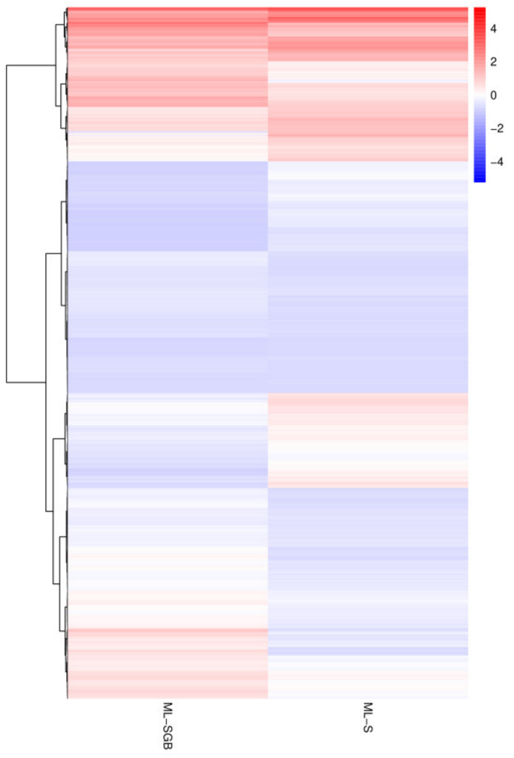

Figure S12 Cluster heatmap of differentially expressed genes between ML-SGB and ML-S

4.Venn Diagrams of Differentially Expressed Genes

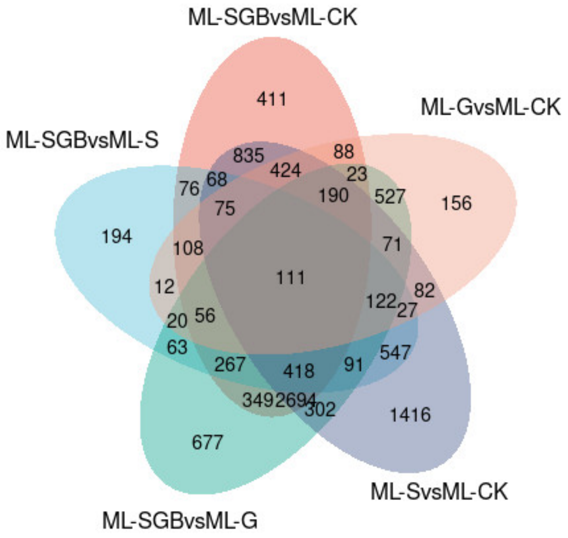

Figure S13 Venn diagram of differentially expressed genes

5.KEGG Pathway Enrichment Analysis

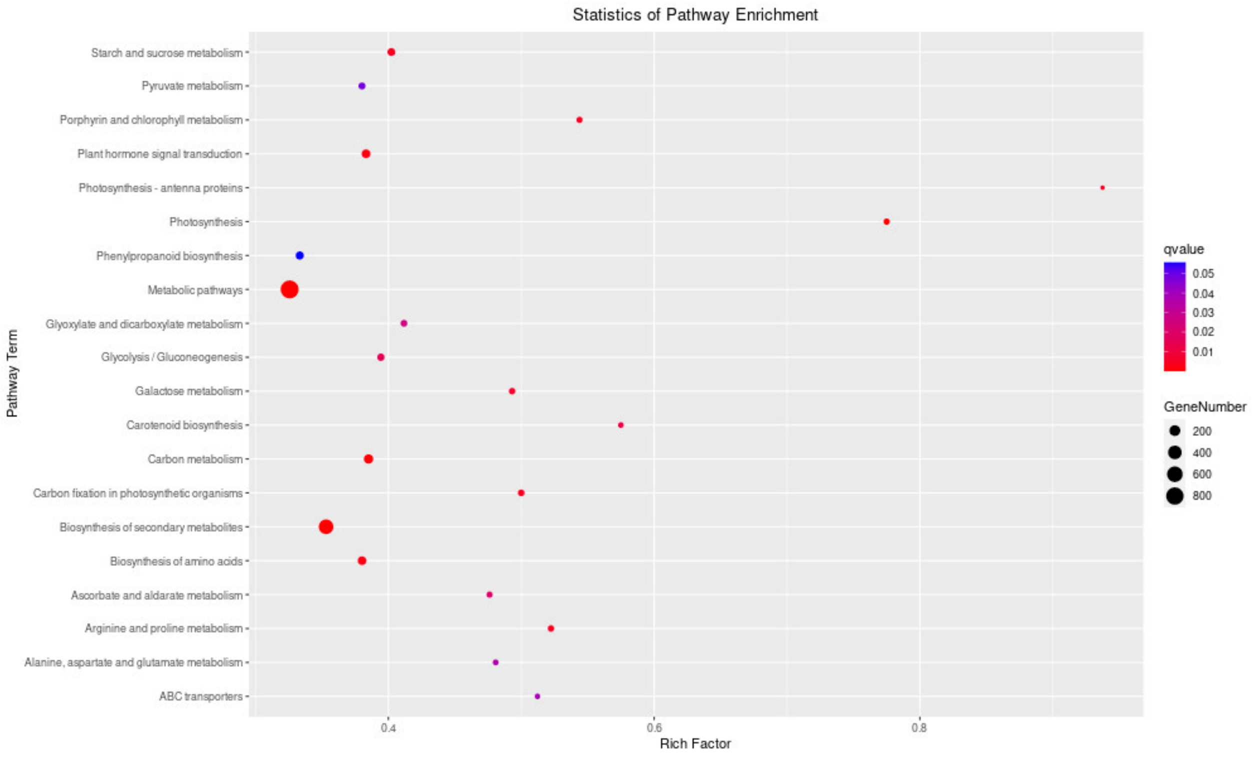

Figure S14 KEGG pathway enrichment analysis between ML-S and ML-CK

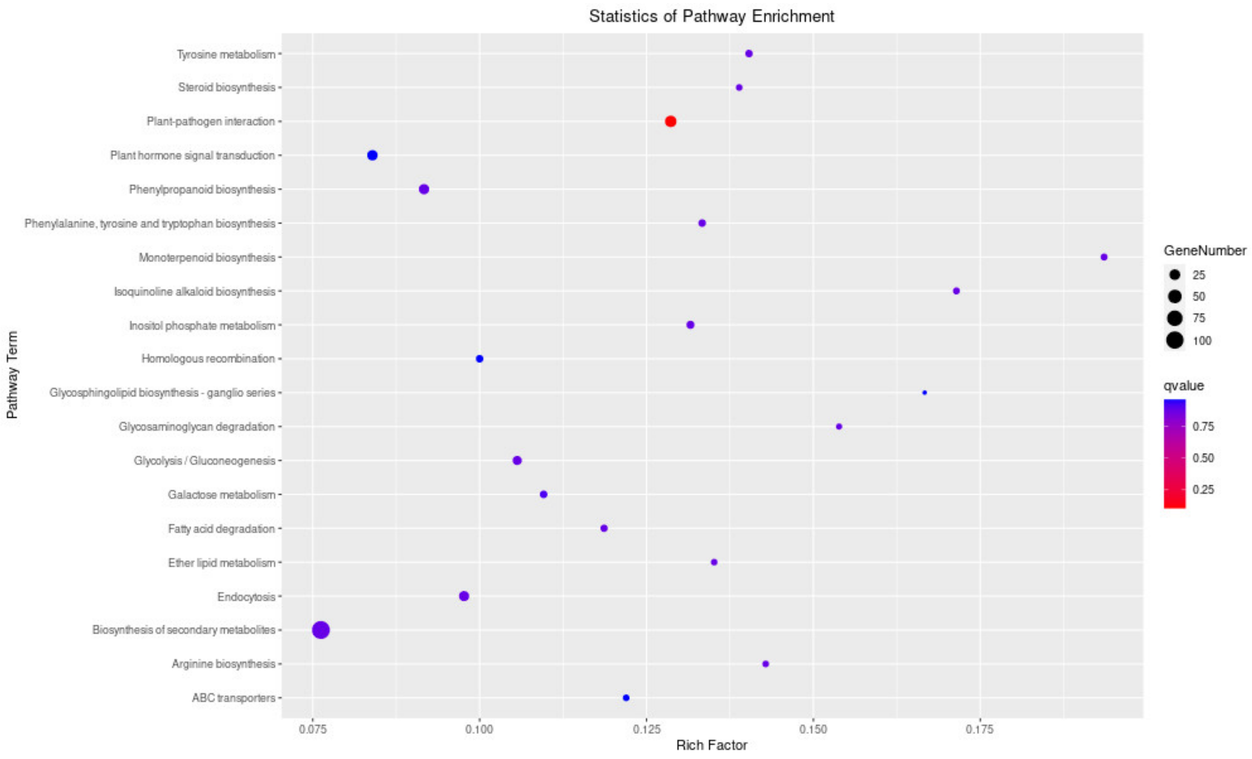

Figure S15 KEGG pathway enrichment analysis between ML-G and ML-CK

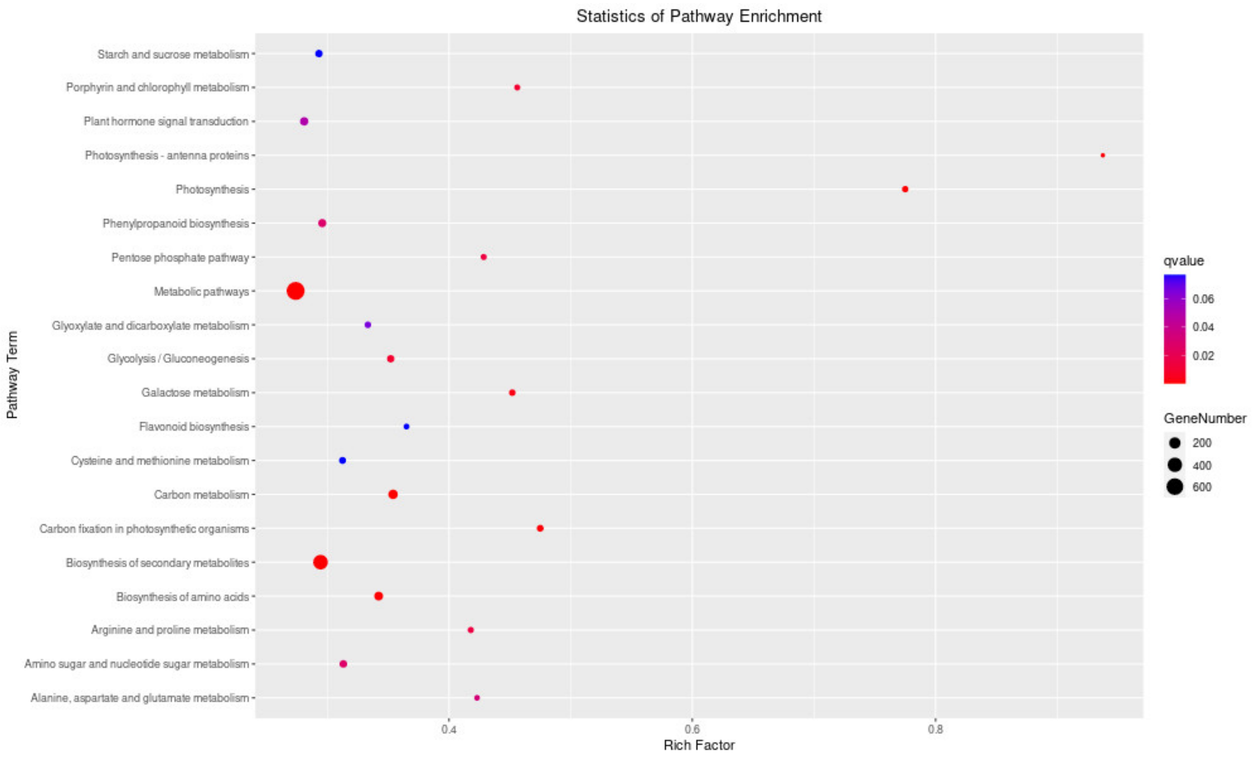

Figure S16 KEGG pathway enrichment analysis between ML-SGB and ML-CK

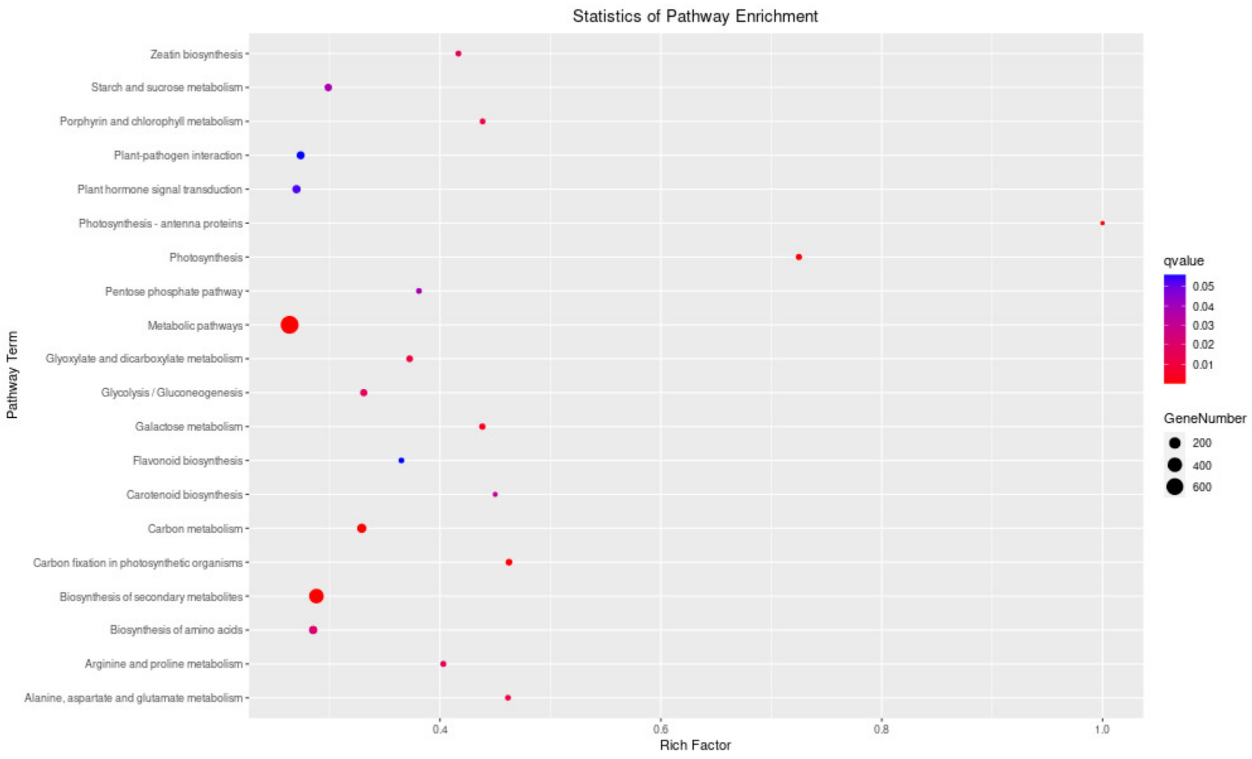

Figure S17 KEGG pathway enrichment analysis between ML-SGB and ML-G

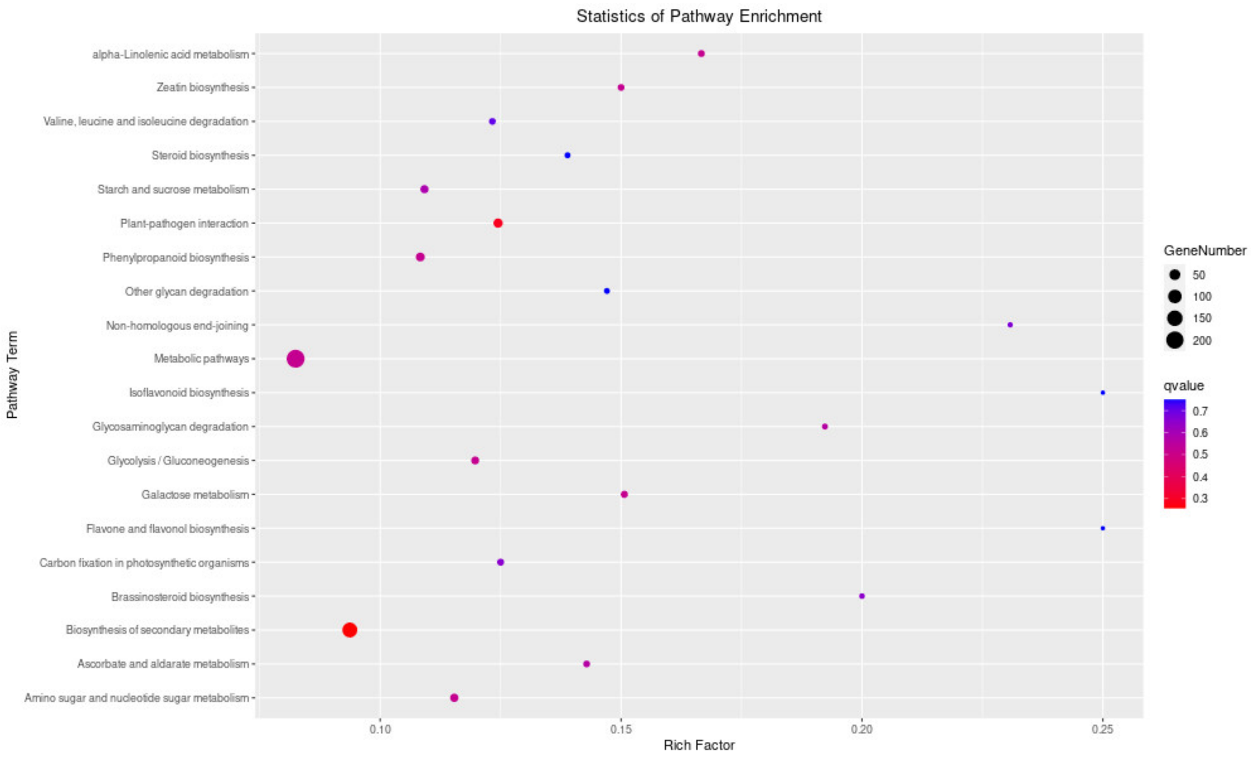

Figure S18 KEGG pathway enrichment analysis between ML-SGB and ML-S

6.GO Functional Classification

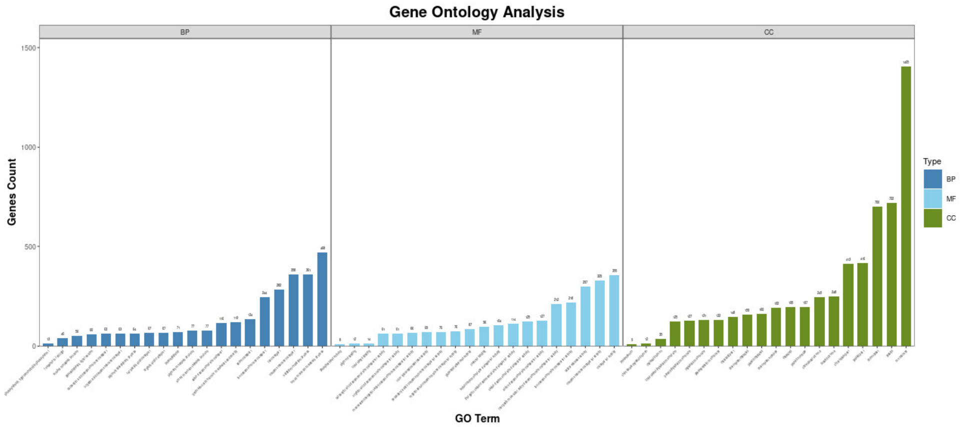

Figure S19 GO functional classification between ML-S and ML-CK

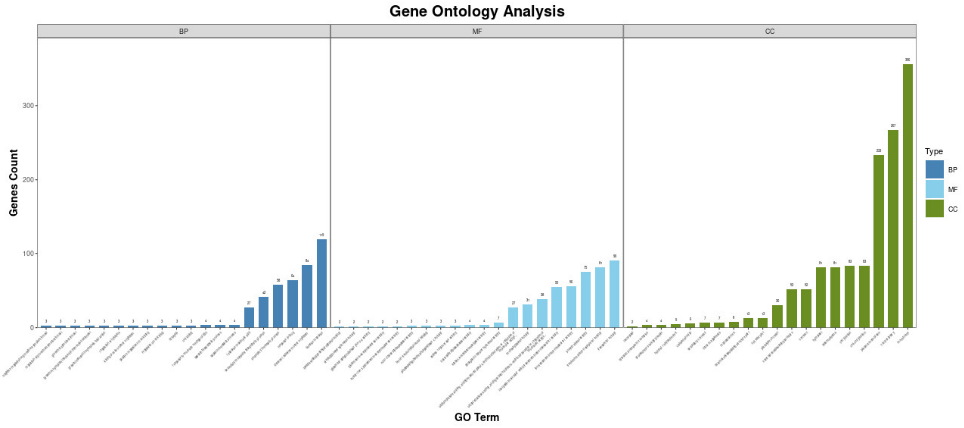

Figure S20 GO functional classification between ML-G and ML-CK

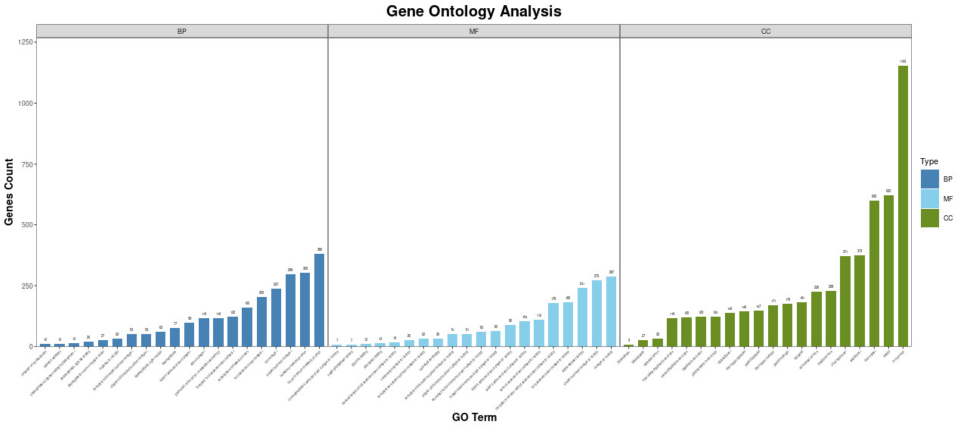

Figure S21 GO functional classification between ML-SGB and ML-CK

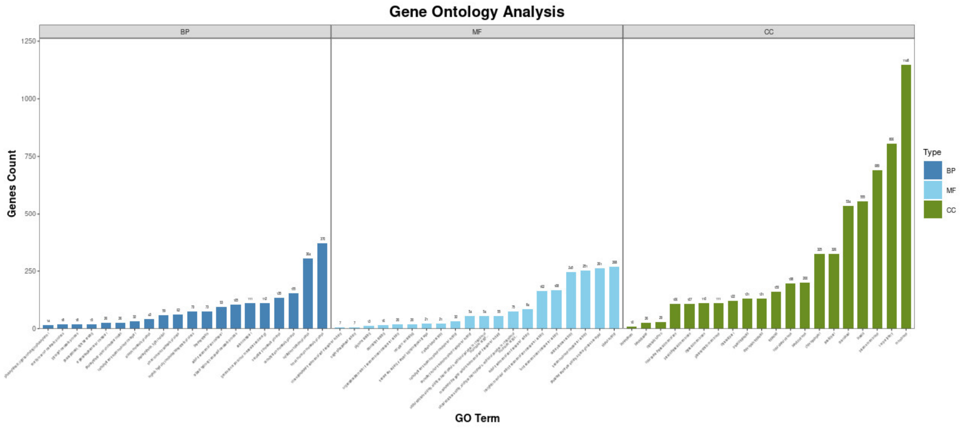

Figure S22 GO functional classification between ML-SGB and ML-G

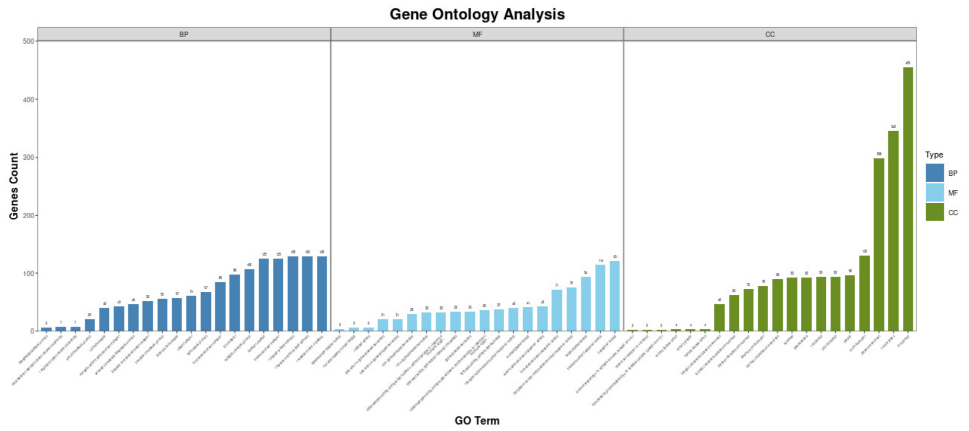

Figure S23 GO functional classification between ML-G and ML-SGB

Supplementary Materials

Table S1. Primer sequences used for qRT-PCR analysis in Quercus acutissima

Gene	Locus / Gene ID	Forward primer (5′–3′)	Reverse primer (5′–3′)
GAPDH	GAPDH	GCTGGTCGTGATCTAACTG	CTTTGCAGTCTCCAACTCCT
SAUR	Hic_asm_3.124	CACCCTGAGTTCCAATGCCT	GCATGGAAGTTAGAGAGCGA
IAA-1	Hic_asm_11.2759	TGGAAGTTGGGTCCAAGAAGG	TGCCACCTCACCTTCACTTC
IAA-3	Hic_asm_7.2228	GATGCTTGTGGGCGATGTTC	TTCTCCATGGCTCTTGGTGC
IAA-2	Hic_asm_4.1261	CCCGACATGAGTGAGGAGTG	ACCCCACTACTTGTGCCTTG
GH3	Hic_asm_6.3024	TTGTTGAGCCCGGAACCTTT	AGTAGCTTGAAACGGCCCTC
PP2C-1	Hic_asm_11.2501	GCGCCGTAGAATCGAGATGA	CCGCCCAGTTAGGATTCTCC
PP2C-2	Hic_asm_5.618	CAGATCACGGTGACGAGGAG	CGAGCCGTACGACAATACCA
PP2C-3	Hic_asm_5.646	AACTGTGGGGATTGTAGGGC	ACCAGCGGCTTCAATTCTCT
PP2C-4	Hic_asm_8.186	TGATTCTGGCGAGTGATGGG	CACGCCTTGTCGGATGTTTC
JAZ	Hic_asm_4.2338	ACCGAACCAGACCCAATCAC	GGCAAAGGCAACCGTTGAG
ERF1	Hic_asm_7.2132	AGTTCCGAAGATGGGTGCTC	ACCACCACATTCTCAACCCT
ALDO-1	Hic_asm_3.88	CCAACCCTCAGTCTCCGTTG	CAAAATTCCACGCCCTGGTG
ALDO-2	Hic_asm_6.3697	GGCAAAGTGAGGAAGAGGCA	AGCCCATGTCTTGAGTGTGC
rpiA	Hic_asm_4.1184	CGCACCTACAGACCCAACTC	ATGCCAGGCTTCACGTACTC
INO1	Hic_asm_7.258	CCTTACCAAGGCCCCTCTTG	TCTCTGGAGCCAATCCAACG
ABCC2	Hic_asm_5.720	CCCACAGGATACCGACAGTG	GACAAGCTGCCCAAACAGTG

Note: Gene names and locus identifiers follow the Quercus acutissima reference genome annotation. GAPDH was used as the internal reference gene. All primers were designed based on the reference genome sequences.
